# Molecular Basis for the Selective Inhibition of Respiratory Syncytial Virus RNA Polymerase by 2'-Fluoro-4'-Chloromethyl-Cytidine Triphosphate

**DOI:** 10.1371/journal.ppat.1004995

**Published:** 2015-06-22

**Authors:** Jerome Deval, Jin Hong, Guangyi Wang, Josh Taylor, Lucas K. Smith, Amy Fung, Sarah K. Stevens, Hong Liu, Zhinan Jin, Natalia Dyatkina, Marija Prhavc, Antitsa D. Stoycheva, Vladimir Serebryany, Jyanwei Liu, David B. Smith, Yuen Tam, Qingling Zhang, Martin L. Moore, Rachel Fearns, Sushmita M. Chanda, Lawrence M. Blatt, Julian A. Symons, Leo Beigelman

**Affiliations:** 1 Alios BioPharma, Inc., South San Francisco, California, United States of America; 2 Department of Pediatrics, Emory University School of Medicine, Atlanta, Georgia, United States of America; 3 Children's Healthcare of Atlanta, Atlanta, Georgia, United States of America; 4 Department of Microbiology, Boston University School of Medicine, Boston, Massachusetts, United States of America; Harvard Medical School, UNITED STATES

## Abstract

Respiratory syncytial virus (RSV) causes severe lower respiratory tract infections, yet no vaccines or effective therapeutics are available. ALS-8176 is a first-in-class nucleoside analog prodrug effective in RSV-infected adult volunteers, and currently under evaluation in hospitalized infants. Here, we report the mechanism of inhibition and selectivity of ALS-8176 and its parent ALS-8112. ALS-8176 inhibited RSV replication in non-human primates, while ALS-8112 inhibited all strains of RSV in vitro and was specific for paramyxoviruses and rhabdoviruses. The antiviral effect of ALS-8112 was mediated by the intracellular formation of its 5'-triphosphate metabolite (ALS-8112-TP) inhibiting the viral RNA polymerase. ALS-8112 selected for resistance-associated mutations within the region of the L gene of RSV encoding the RNA polymerase. In biochemical assays, ALS-8112-TP was efficiently recognized by the recombinant RSV polymerase complex, causing chain termination of RNA synthesis. ALS-8112-TP did not inhibit polymerases from host or viruses unrelated to RSV such as hepatitis C virus (HCV), whereas structurally related molecules displayed dual RSV/HCV inhibition. The combination of molecular modeling and enzymatic analysis showed that both the 2'F and the 4'ClCH_2_ groups contributed to the selectivity of ALS-8112-TP. The lack of antiviral effect of ALS-8112-TP against HCV polymerase was caused by Asn291 that is well-conserved within positive-strand RNA viruses. This represents the first comparative study employing recombinant RSV and HCV polymerases to define the selectivity of clinically relevant nucleotide analogs. Understanding nucleotide selectivity towards distant viral RNA polymerases could not only be used to repurpose existing drugs against new viral infections, but also to design novel molecules.

## Author Summary

Viral RNA polymerase complexes mediate all of the enzymatic functions required for genomic replication and transcription in RNA viruses. Because of their essential role in the virus life cycle, RNA polymerases are major molecular targets for antiviral therapies. Sofosbuvir and mericitabine are related compounds belonging to a class of drugs called nucleoside analogs that inhibit the RNA polymerase of hepatitis C virus (HCV), a positive-strand RNA virus, but have no effect on negative-strand RNA viruses. The mechanistic reason for this inactivity is unknown. The only nucleoside analog currently under clinical evaluation against respiratory syncytial virus (RSV), a negative-strand RNA virus, is ALS-8176. In this study, we present the detailed mechanism of action of ALS-8112, the parent molecule of ALS-8176. A multidisciplinary approach combining cellular, chemical, structural, and enzymatic methods was employed to demonstrate that the triphosphate form of ALS-8112 targets the RNA polymerase of RSV, but not of HCV. A series of molecules structurally related to ALS-8112 displayed dual RSV/HCV inhibition, whereas mericitabine only targeted HCV RNA polymerase. Understanding the molecular basis of nucleotide selectivity towards distant viral RNA polymerases could not only be used to repurpose existing drugs against new viral infections, but also to design novel molecules with broad antiviral spectrum.

## Introduction

Respiratory syncytial virus (RSV) is a non-segmented, single-stranded, negative sense RNA virus and a member of the family *Paramyxoviridae*, which also includes human metapneumovirus and parainfluenza virus type-3 (PIV-3). RSV infection and resulting clinical sequelae usually last 1–2 weeks and results in mild “cold-like” symptoms in the majority of adults. However, RSV is an important pathogen in the elderly, immunocompromised patients, and patients with cardiopulmonary disease [1,2]. RSV is also a leading cause of lower respiratory disease in infants [3,4]. In 2005, an estimated 33.8 million episodes of RSV infection occurred worldwide in infants and young children with most of these occurring in otherwise healthy full-term infants. Of these, at least 3.4 million severe cases of lower respiratory tract infection (LRI) required hospitalization, and an estimated 66,000 to 199,000 deaths occurred, mostly in the developing world [3]. In addition to the acute morbidity and mortality associated with RSV infection, LRI due to RSV may have long-term consequences as it has also been strongly associated with and implicated as a cause of childhood asthma [5]. Risk factors for severe illness associated with RSV infection include prematurity (≤ 35 weeks gestation) and younger age (under 2 years) [6], pulmonary deficiencies, congenital heart disease, immunosuppression, low birth weight, large family size, exposure to passive smoke, and lack of breast feeding [7].

No vaccines are available for the prevention of RSV infection. Palivizumab, a monoclonal antibody directed against RSV, is approved in the United States as a prophylactic for the prevention of serious lower respiratory tract disease caused by RSV in children at high risk of RSV. However, prophylaxis is only effective in preventing hospitalization in approximately 50% of individuals and the cost is prohibitive for otherwise healthy infants and for children in developing countries. Treatment of infants with severe RSV bronchiolitis is limited to supportive oxygen therapy and fluids. Aerosolized ribavirin, a base-modified guanosine nucleoside analog and broad-spectrum antiviral agent, is approved for hospitalized infants and young children with severe LRIs, but its use is limited due to uncertain efficacy and complexity of administration. Despite a clear unmet medical need, only a few therapeutic agents have reached clinical development [8]. These agents are primarily viral fusion inhibitors that, like neutralizing antibodies, may prevent the spread of infection from already infected cells but have no effect on intracellular viral replication [9]. A recent proof-of-concept study demonstrated that in healthy adults challenged with RSV, treatment with the fusion inhibitor, GS-5806, reduced the viral load and the severity of clinical disease [10]. Although this study represents an important step forward in the development of RSV therapeutics, mechanisms of action involving an extracellular step may have intrinsic limitations for therapeutic applications against acute infections due to a potentially reduced window of intervention. Another potential limitation to the use of fusion inhibitors is the rapid emergence of drug-selected resistance-associated variants yielding pathogenic RSV variants that are cross-resistant to this class of molecules [11]. For these reasons, and due to the scarcity of clinical data from therapeutic candidates used against RSV, the possibility of effectively treating naturally occurring RSV infection with a small molecule remains an unanswered question.

With over 25 drugs approved for the treatment of serious viral diseases, nucleoside and nucleotide analogs represent the largest class of antiviral drugs. As most of these molecules were developed to treat DNA viruses such as herpesvirus, HIV, or HBV, nucleoside analogs are generally considered to be most beneficial in suppressing chronic infections. Antiviral nucleosides such as zidovudine (AZT) or lamivudine (3TC) share the same mechanism of action—inhibition of viral DNA polymerases through their triphosphate form, causing chain termination after incorporation of the nucleotide to the DNA [12]. The hepatitis C virus (HCV) inhibitor sofosbuvir is the first FDA-approved antiviral of this class to inhibit a viral RNA polymerase. Unfortunately, there are currently no examples of ribonucleoside analogs that are clinically efficacious against RNA viruses other than HCV [13]. Favipiravir (T-705) and BCX4430 are two broad-spectrum nucleoside analogs currently under evaluation in Ebola-infected patients based on encouraging animal efficacy [14–16], but their precise mechanism of action and degree of selectivity towards viral and host targets remain unclear [16,17].

The small molecule ALS-8176 (also referred to as ALS-008176) is a first-in-class nucleoside analog prodrug currently undergoing clinical evaluation for the treatment of RSV infection in hospitalized infants (ClinicalTrials.gov identifier: NCT02202356). ALS-8176 and its parent cytidine analog ALS-8112 were recently discovered to be potent inhibitors of RSV replication in vitro [18]. In clinical trials, ALS-8176 given orally was efficacious against RSV infection in adult volunteers [19]. In this paper, we present the mechanism of action and the antiviral selectivity of ALS-8176 and ALS-8112. We show that ALS-8176 inhibited RSV replication in non-human primates while ALS-8112 inhibits a broad panel of RSV A and B subtypes in vitro, as well as RSV-related viruses from the *Paramyxoviridae* and the *Rhabdoviridae* families. We identify the RNA polymerase function of the L protein of RSV as the molecular target of ALS-8112 by selecting and characterizing drug resistance-associated mutations. In enzymatic assays, we show that the 5'-triphosphate form of ALS-8112 (ALS-8112-TP) causes immediate chain termination of RNA synthesis and inhibition of the viral polymerase activity, a hallmark of many approved antiviral nucleoside analogs. Finally, we provide a mechanistic basis for target selectivity by evaluating clinically-relevant ribonucleotide analogs that specifically inhibit the RNA polymerase of RSV, HCV, or both. We find that subtle structural changes in nucleotides dramatically alter their antiviral spectrum. The potential medical implication of these findings is discussed.

## Results

### ALS-8112 is a pan-strain inhibitor of RSV replication in vitro

**Fig 1:**
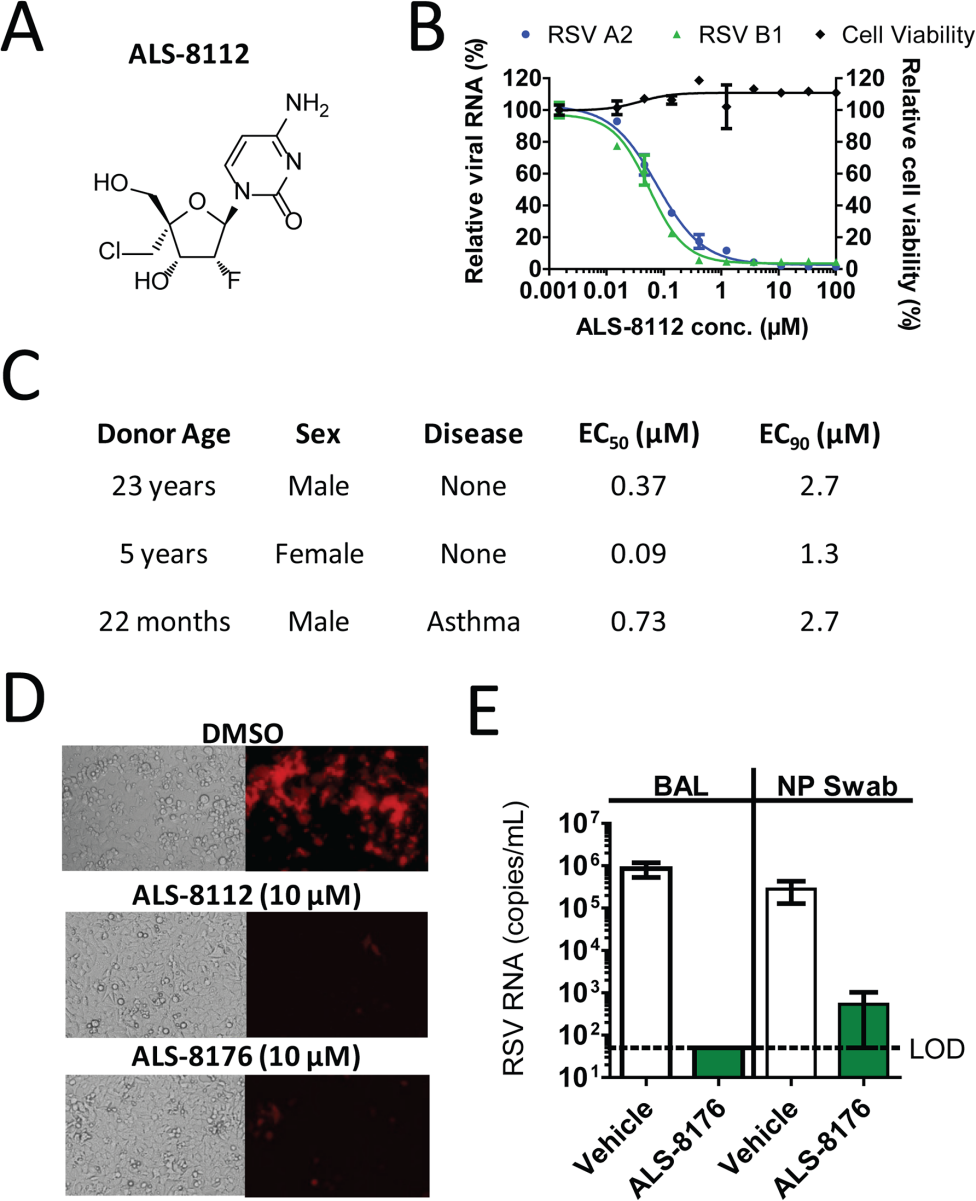
Inhibition of RSV replication by ALS-8112 and ALS-8176. (**A**) Chemical structure of 2'-fluoro-4'-chloromethyl (2'F-4'ClCH2) cytidine, or ALS-8112. (**B**) In vitro inhibition potency of ALS-8112 against the RSV A2 and B1 strains grown in HEp-2 cells. The viral RNA level was measured by qRT-PCR, and reported as percentage of the uninhibited condition (n = 3). The effect of ALS-8112 on the viability of human epithelial HEp-2 cells was also evaluated. The highest concentration of ALS-8112 used to measure the concentration resulting in 50% cytotoxicity (CC50) was 100 μM (n = 3). (**C**) In vitro efficacy of ALS-8112 in a three-dimensional lung model. Primary human tracheal/bronchial epithelial cells from three individual human donors were infected on the apical side with the RSV A2 strain, while increasing concentrations of ALS-8112 were added to the basal medium. (**D**) Fluorescence microphotographs of HEp-2 cells infected with recombinant RSV-mKate2, in the presence of DMSO, 10 μM ALS-8112, or 10 μM ALS-8176. (**E**) African Green monkey efficacy model. ALS-8176 was administered BID for a total of 6 days. At the end of treatment (Day 5 post-infection), RSV RNA titers were measured in bronchoalveolar lavage (BAL) and nasopharyngeal (NP) swab samples for each group (vehicle and drug) containing four animals. Limit of detection (LOD) for qRT-PCR analysis was 50 copies/mL (dashed line).

A series of ribonucleoside analogs was recently identified as inhibiting the replication of RSV, and the optimization of the precursor molecules led to the chemical synthesis of 2'-fluoro-4'-chloromethyl (2'F-4'ClCH_2_) cytidine, referred to as ALS-8112 (Fig 1A) [18]. The nucleoside analog ALS-8112 did not significantly decrease the viability of human epithelial (HEp-2) cells after 5 days (with the concentration resulting in 50% cytotoxicity [CC_50_] > 100 μM) (Fig 1B). Using the same cell type and assay duration, ALS-8112 inhibited the RNA replication of RSV A2 and B1 strains with concentration resulting in 50% inhibition (EC_50_) values of 0.153 ± 0.076 μM, and 0.132 ± 0.055 μM, respectively (Fig 1B). In addition, ALS-8112 demonstrated potent inhibition of a range of diverse RSV clinical isolates with comparable EC_50_ values (Table A in S1 Text). To understand the role of 5'-triphosphate formation in antiviral effect, we synthesized ALS-8112-I, an analog of ALS-8112 in which the 5'-hydroxyl group was replaced by iodine (Fig AA in S1 Text). Because of this modification, ALS-8112-I cannot form any triphosphate in vitro. As expected, ALS-8112-I did not significantly inhibit the luciferase activity in the RSV replicon (Fig AB in S1 Text). The in vitro antiviral activity of ALS-8112 was also characterized in a three-dimensional tissue culture system. This system consists of normal, human-derived tracheal/bronchial epithelial cells cultured to form a pseudo-stratified cell arrangement closely resembling the epithelial tissue of the respiratory tract [20,21]. The apical surface of the cultures contains numerous microvilli and cilia and the presence of tight junctions resembles the normal epithelial tissue of the lung. In this in vitro three-dimensional lung model, ALS-8112 was added to the basal media and incubated overnight before RSV strain A2 was added to the apical side of the system. The antiviral activity of ALS-8112 in human donor cells (n = 3) is described in Fig 1C and Fig B in S1 Text. In the donor cells, ALS-8112 inhibited RSV RNA replication with an EC_50_ ranging between 0.09 and 0.73 μM, and 90% inhibition (EC_90_) between 1.3 and 2.7 μM. We conclude that ALS-8112 is a pan-strain inhibitor of RSV replication in vitro, and that the antiviral activity of ALS-8112 is dependent upon the formation of its 5'-triphosphate metabolite.

### ALS-8176 inhibits RSV replication in vitro and in vivo

We have previously shown that ALS-8112 and ALS-8176 inhibit RSV replication in a subgenomic luciferase-based replicon assay [18]. This effect also correlated with changes in viral protein synthesis, as judged by the reporter red fluorescent protein produced by recombinant RSV infectious particles (Fig 1D). Because of its high oral bioavailability [18], the 2',3’-diester prodrug ALS-8176 was evaluated for in vivo efficacy in non-human primates. The effect of oral treatment with ALS-8176 on the replication of RSV A2 in the African Green monkey model was studied. ALS-8176 was administered twice daily (BID), starting with a single loading dose of 200 mg/kg one day prior to RSV inoculation and followed by maintenance doses of 50 mg/kg given BID for a total treatment duration of 6 days. Based on the pharmacokinetic profile of ALS-8176 [18], this dose was predicted to deliver an intracellular level of ALS-8112-TP in the lung of ~5-fold the level required to achieve the antiviral EC_90_ *in vitro*. At the end of treatment, RSV RNA titers reached approximately 1 × 10^6^ copies/mL in bronchoalveolar lavage (BAL) samples from the four animals dosed with vehicle. In contrast, in all four ALS-8176-treated animals, RSV RNA was undetectable (< 50 copies/mL) in samples collected (Fig 1E). This represents a difference of > 4 log_10_ copies/mL between the RSV RNA titers of the vehicle-treated animals and those of the ALS-8176-treated animals indicating that ALS-8176 significantly suppressed RSV replication in vivo. A similar profile was observed in nasopharyngeal (NP) swab samples (Fig 1E), although in this case the difference in RSV RNA titers between the vehicle and ALS-8176-dosed groups was about 3 log_10_ copies/mL. These results demonstrate that ALS-8176 significantly inhibits RSV replication in vivo.

### The L protein of RSV is the molecular target of ALS-8112

**Fig 2:**
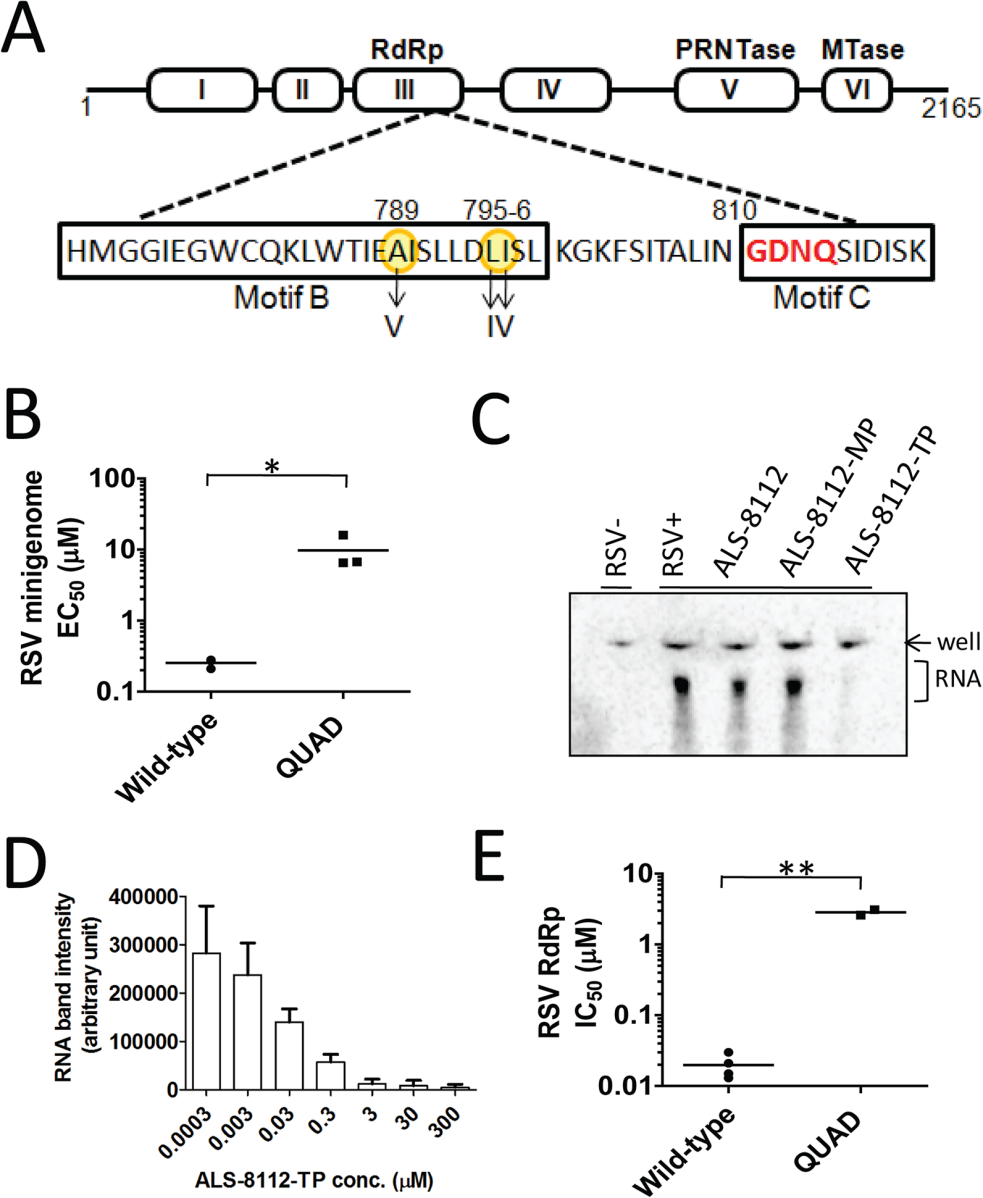
Identification of RSV polymerase as the molecular target for ALS-8112. (**A**) Selection of resistance mutations associated with the prolonged culture of RSV A2 in the presence of increasing concentrations of ALS-8112. All four mutations detected from full-genome sequence analysis mapped to motif B, a region of the RdRp domain (residues ~500–1100) of the RSV L protein just upstream of the CRIII region containing the catalytic motif _810-_GDNQ_-813_ (red) responsible for nucleotide incorporation by the RSV polymerase. (**B**) In vitro inhibition potency of ALS-8112 against the RSV minigenome luciferase-based reporter assay. HEp-2 cells were co-transfected to transiently express the RSV N, P, M2-1 and L proteins containing either the wild-type or the QUAD mutated sequence (n = 3) *P < 0.05 (Student's *t* test). (**C**) Inhibitory effect of ALS-8112-TP on the RdRp activity of the crude RSV RNP complex. The RNP complex containing the RSV L protein was extracted from virus-infected cells (RSV+), and uninfected cells were used as negative control for RdRp activity (RSV-). The enzymatic reaction was conducted in the presence of ALS-8112, ALS-8112-MP, or ALS-8112-TP. Labeled transcripts were separated from the initial radiolabeled CTP substrate by urea PAGE prior to phosphor-imaging. (**D**) Effect of increasing concentration of ALS-8112-TP on the RdRp activity of the RNP complex. The intensity of RNA product after gel electrophoresis was reported for each nucleotide concentration (n *= 3*). (**E**) In vitro inhibition potency of ALS-8112-TP against RSV wild-type (n = 4) and QUAD (n = 2) RdRp activity **P < 0.005 (Student's *t* test).

RSV A2 viruses were repeatedly passaged in HEp-2 cells with or without increasing concentrations of ALS-8112. Two independent adaptations were conducted, as well as a no-drug control virus pool that was passaged at the same time. After > 35 passages, the virus grown in the presence of ALS-8112 from each ALS-8112-treated pool exhibited a > 50-fold EC_50_ shift in the antiviral assay when compared with the control virus pool cultured for the same duration in the absence of ALS-8112. When the complete RSV genome was sequenced, all ALS-8112-selected viruses showed four amino acid substitutions (QUAD); methionine 628 to leucine (M628L), alanine 789 to valine (A789V), leucine 795 to isoleucine (L795I), and isoleucine 796 to valine (I796V) within the CRIII of the RdRp, the RNA polymerase coding region of the RSV L gene (Fig 2A). Within the ALS-8112 drug selection pool, >95% of the viruses carried all four mutations on the same genome. Three of the four amino acid changes associated with ALS-8112 resistance are co-located within motif B. This particular sequence of the CRIII region is in close proximity to the catalytic motif C residues (_810-_GDNQ_-813_) that are responsible for nucleotide incorporation by paramyxovirus RNA polymerases. No mutations were identified in any other RSV genes. Phenotypic validation of the QUAD mutations was conducted by reverse genetics using the RSV minigenome system. In this assay, four plasmids encoding RSV N, P, M2-1, and L proteins were co-transfected into HEp-2 cells. The transient expression of each of the corresponding proteins leads to the formation of a functional RSV polymerase complex, and its RNA-dependent RNA polymerase (RdRp) activity was monitored in the cells using a luciferase-based reporter [22]. We mutated the L gene at all four positions previously identified from sequencing, and analyzed the effect of these substitutions on the inhibition potency of the drug. In this system, ALS-8112 inhibited the wild-type RSV polymerase-dependent luciferase activity with an EC_50_ of 0.25 ± 0.04 μM (Fig CA in S1 Text). In comparison, ALS-8112 inhibited the QUAD-mutant RSV polymerase-dependent luciferase activity with an EC_50_ of 9.7 ± 5.4 μM, only reaching about 60% maximum inhibition at the top concentration (Fig 2B and Fig CA in S1 Text). The presence of the four amino acid changes did not significantly affect the amount of firefly luciferase signal, which may indicate similar RSV polymerase activity between the two clones (Fig CB in S1 Text). The 39-fold shift in inhibition potency caused by the four mutations in the L gene indicates that ALS-8112 inhibits RSV replication by targeting the L protein responsible for the viral RNA transcription and replication functions. A detailed description of the characterization of the ALS-8112 resistant virus and the role of each individual mutation will be the subject of a separate study.

### Inhibition of wild-type and mutant RSV polymerase by ALS-8112-TP

Crude ribonucleoprotein (RNP) complex containing the RSV L protein was extracted from virus-infected cells. The RdRp activity was specific to the viral RNP complex, and was not found in uninfected cells (Fig 2C). The viral RNA transcription activity was inhibited by ALS-8112-TP, but not by ALS-8112 5'-monophosphate (ALS-8112-MP), nor by the non-phosphorylated parent nucleoside, ALS-8112. The RNA transcription activity of the RSV–RNP complex was dose-proportionally inhibited by ALS-8112-TP with an IC_50_ of 0.020 ± 0.008 μM (Fig 2D and Fig CC in S1 Text). The same RNP extraction was performed with cells infected with the QUAD mutated virus. The RdRp activity of the QUAD RNP complex was only 57% inhibited by saturating concentrations of ALS-8112-TP, with an IC_50_ of 2.9 ± 0.4 μM (Fig CD in S1 Text). This corresponds to a 145-fold loss in inhibition potency compared to the wild-type protein (Fig 2E and Fig CE in S1 Text).

**Fig 3:**
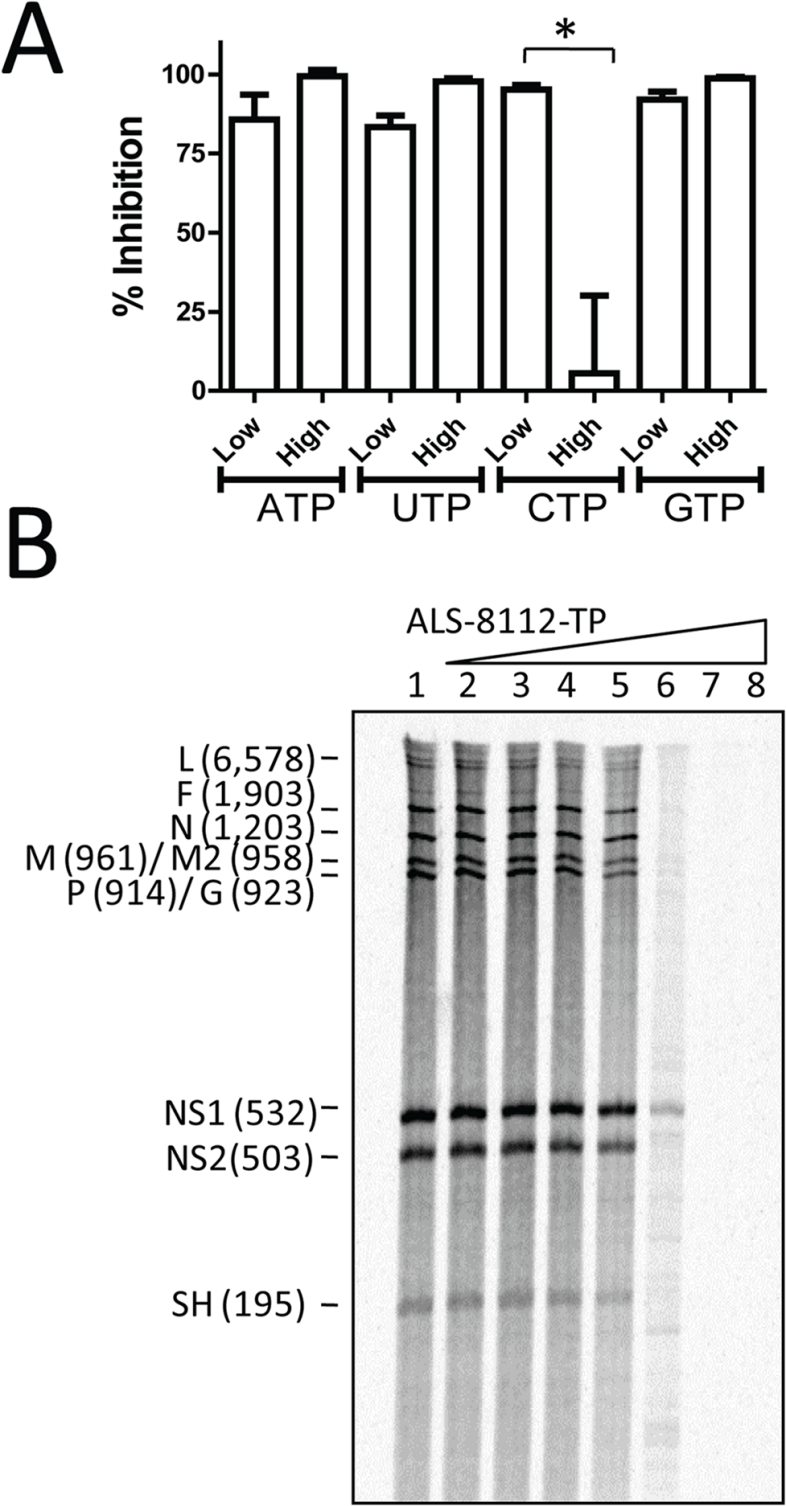
Competitive inhibition of RSV RNP complex by ALS-8112-TP. (**A**) Effect of low (1 μM) and high (100 μM) concentration of ATP, UTP, CTP, or GTP, on inhibition of RSV RNP by ALS-8112-TP used at a single concentration of 30 μM. (n = 2) *P < 0.05 (Student's *t* test). (**B**) Effect of increasing concentration of ALS-8112-TP on the RdRp activity of the RNP complex. RNA products were treated with RNase H prior to high-resolution electrophoresis in order to visualize individual gene transcripts. Lane 1 had no inhibitor, and ALS-8112-TP concentration ranged from 0.0003 μM (lane 2) to 300 μM (lane 8) by 10-fold increments.

The inhibition of RNA synthesis catalyzed by the wild-type RNP complex in the presence of ALS-8112-TP was inversely proportional to the concentration of cytidine triphosphate (CTP) in the reaction (Fig 3A). However, the concentration of adenosine triphosphate (ATP), guanosine triphosphate (GTP), and uridine triphosphate (UTP) had no effect on the inhibition potency of ALS-8112-TP. Therefore, ALS-8112-TP is a competitive inhibitor of cytidine monophosphate (CMP) incorporation into nascent RNA product by the RSV RNP complex. To understand the effect of ALS-8112-TP on the production of RNA transcripts by crude wild-type RNP complex, products of RNA synthesis were treated with RNase H and visualized by high-resolution gel electrophoresis [23]. Using this method, individual gene transcripts were separated, and their inhibition correlated with the increase in concentration of ALS-8112-TP (Fig 3B).

### The antiviral spectrum of ALS-8112 correlates with the selectivity of ALS-8112-TP towards polymerases

Since ALS-8112 inhibited all tested lab-adapted and clinical strains of RSV equally well, the potential for cross-species antiviral activity was evaluated in infected cells. ALS-8112 also inhibited the replication of RSV-related PIV-3 from the *Paramyxoviridae* family, as well as the more distant vesicular stomatitis virus from the *Rhabdoviridae* family (Table 1, and Fig D in S1 Text). However, ALS-8112 did not inhibit unrelated representative viruses with negative-strand segmented RNA or with positive-strand RNA genomes such as influenza A and HCV, respectively (Table 1, and Fig D in S1 Text). This trend in pan-antiviral profile limited to non-segmented ssRNA(-) viruses converged with the selectivity of ALS-8112-TP towards viral RNA polymerases. In enzymatic assays, ALS-8112-TP inhibited the RdRp activity of PIV-1 polymerase, but not of RSV-unrelated polymerases from influenza A or HCV (Table 2, and Fig E in S1 Text). When tested for selectivity against human enzymes, ALS-8112-TP was not recognized as a substrate for human mitochondrial RNA polymerase (Fig F in S1 Text, and [18]). In comparison, 4'N_3_-CTP, the triphosphate form of balapiravir, was efficiently incorporated into the RNA by the mitochondrial enzyme (Fig F in S1 Text, and [24]). 2'F-CTP, but not 4'ClCH_2_-CTP, was also recognized as a substrate. Therefore, the discrimination of ALS-8112-TP by the mitochondrial polymerase was provided mainly by its 4'ClCH_2_ moiety. We conclude that ALS-8112-TP is a potent and selective chain terminator of RSV and other *Paramyxoviridae* RNA polymerases, with low potential for interaction with human polymerases.

**Table 1:** Inhibition potency of ALS-8112 against RSV and other RNA viruses.

Virus	Family	Group	EC_50_ (μM)	CC_50_ * (μM)
Respiratory syncytial virus	*Paramyxoviridae*	Non-segmented ssRNA (-)	0.15	> 100 ^(a^ ^)^
Parainfluenza virus type-3	*Paramyxoviridae*	Non-segmented ssRNA (-)	1.3	> 100 ^(b)^
Vesicular stomatitis virus	*Rhabdoviridae*	Non-segmented ssRNA (-)	3.4	> 100 ^(b)^
Influenza virus	*Orthomyxoviridae*	Segmented ssRNA (-)	> 100	> 100 ^(b)^
Hepatitis C virus	*Flaviviridae*	ssRNA (+)	> 100	> 100 ^(c)^
Rhinovirus 1b	*Picornaviridae*	ssRNA (+)	> 100	> 100 ^(^ ^d)^

*CC_50_ values for ALS-8112 were obtained using HEp-2

^(a)^, A549

^(b)^, Huh-7

^(c)^, and HeLa-Ohio

^(d)^ cells.

**Table 2:** Inhibition potency of ALS-8112-TP against RSV and other viral RNA polymerases.

Virus	Family	Group	IC_50_ (μM)
Respiratory syncytial virus	*Paramyxoviridae*	Non-segmented ssRNA (-)	0.02
Parainfluenza virus type-1	*Paramyxoviridae*	Non-segmented ssRNA (-)	2.3
Influenza virus	*Orthomyxoviridae*	Segmented ssRNA (-)	> 100
Hepatitis C virus	*Flaviviridae*	ssRNA (+)	> 33

### Chain termination of RNA synthesis by recombinant RSV polymerase

**Fig 4:**
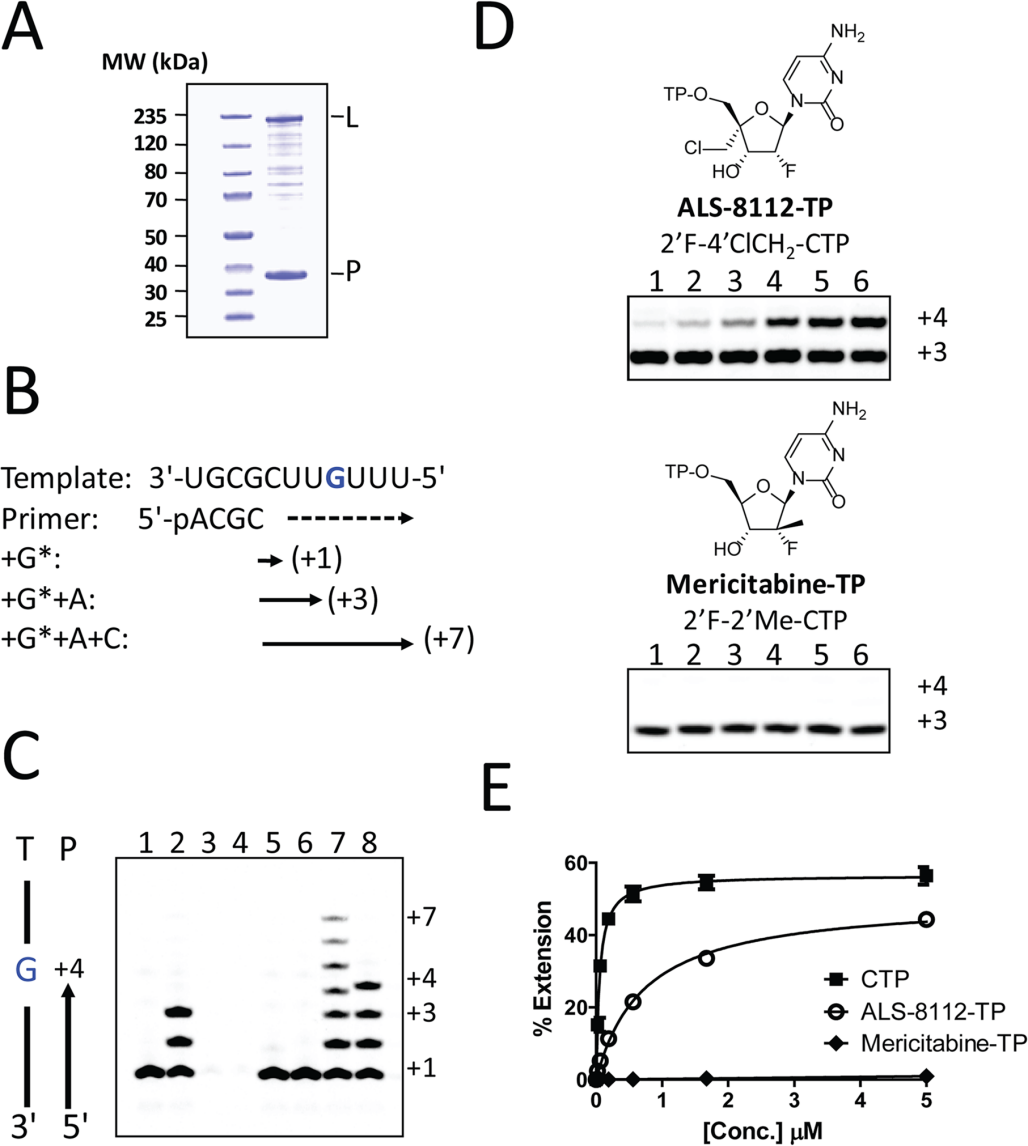
Chain termination of RNA synthesis by ALS-8112-TP. **(A)** SDS PAGE of recombinant RSV L-P polymerase complex. (**B**) Principle of the single nucleotide incorporation assay with RSV L-P polymerase: The 11-mer template contains a single G at the +4 position. In presence of radiolabled GTP (G*), the primer can be extended by one base (+1). GTP+ATP allows for a +3 extension, while the addition of CTP enables full-length RNA product synthesis (+7). (**C**) Wild-type recombinant RSV L-P polymerase complex (all lanes except 3 and 4) was incubated with primer/template (P/T) and GTP* (lane 1), or GTP* + 10 μM ATP (lane 2). Lanes 3 and 4: same as 1 and 2, except that the lethal N812A mutation was introduced within the L subunit. Lane 5: enzyme + GTP* + 10 μM CTP. Lane 6: enzyme + GTP* + 10 μM ALS-8112-TP. Lane 7: enzyme + GTP* + ATP + 10 μM CTP. Lane 8: enzyme + GTP* + ATP + 10 μM ALS-8112-TP. (**D**) RSV L-P incubated in the presence of GTP* + ATP and increasing concentrations of either ALS-8112-TP or mericitabine-TP: lane 1, 0.021 μM; lane 2, 0.062 μM; lane 3, 0.19 μM; lane 4, 0.56 μM; lane 5, 1.7 μM; and lane 6, 5.0 μM. (**E**) Product formation was quantified and expressed as % primer extension from the +3 position (see calculation in Fig D in S1 Text). CTP K_m_ = 0.057±0.009 μM (*n* = 4), and ALS-8112-TP K_m_ = 0.74±0.08 μM (*n* = 4).

Recently, it has been shown that, once co-purified, recombinant RSV L and P proteins form a dimer that recognizes synthetic RNA templates to synthesize short products (Fig 4A, [25]). In a similar assay format, the RNA polymerase activity of recombinant RSV L-P polymerase in complex with a short synthetic primer/template substrate was monitored (Fig 4B **)**. In the presence of GTP alone or GTP+ATP, wild-type RSV L-P RNA polymerase complex specifically extended the RNA primer by 1 or 3 bases, respectively (Fig 4C, lanes 1 and 2). As a control, the L-P protein variant containing a single N812A mutation within the catalytic site of the RdRp region of L was inactive (lanes 3 and 4). Under these conditions, no signs of cytidine mis-incorporation were observed in the absence of ATP (lane 5). However, the specific incorporation of natural CMP in the presence of GTP and ATP led to the formation of +7 full-length RNA products (lane 7). In comparison, ALS-8112-MP was also incorporated into the nascent RNA at a specific +4 position opposite to a single guanosine on the template (lane 8), but not at any other positions (lane 6). After the incorporation of ALS-8112-MP, no subsequent nucleotide could be incorporated at the 3ʹ-end of the RNA primer and full-length product formation (+7 position) was not achieved (lane 8). This finding demonstrates that ALS-8112-TP inhibits the polymerase activity of the RSV L-P RNA polymerase complex by immediate termination of chain synthesis. The incorporation of ALS-8112-MP by RSV polymerase was efficient, as judged by the modest 13±2.5-fold discrimination relative to natural CTP (Fig 4D and 4E). In comparison, the specific inhibitor of HCV RNA polymerase mericitabine-TP (2'F-2'Me-CTP) was not recognized as a substrate for RSV polymerase under standard assay conditions. Even at a concentration of 300 μM, the level of incorporation of mericitabine-MP was less than 20% (Fig G in S1 Text). Compared to natural CTP, this represents a discrimination level greater than 5,000-fold. Consistently, mericitabine did not inhibit RSV replication in the subgenomic replicon assay (Table 3).

**Table 3:** Inhibition potency of cytidine analogs against RSV versus HCV replicon.

Compound	RSV EC_50_ (μM)	HCV EC_50_ (μM)
2'F-4'ClCH_2_-cytidine (ALS-8112)	0.15	> 100
2'F-2'Me-cytidine (mericitabine)	> 100	0.85
4'N_3_-cytidine (balapiravir)	0.67	0.84

### Molecular basis for the selectivity of nucleotide analogs toward RNA polymerases

**Fig 5:**
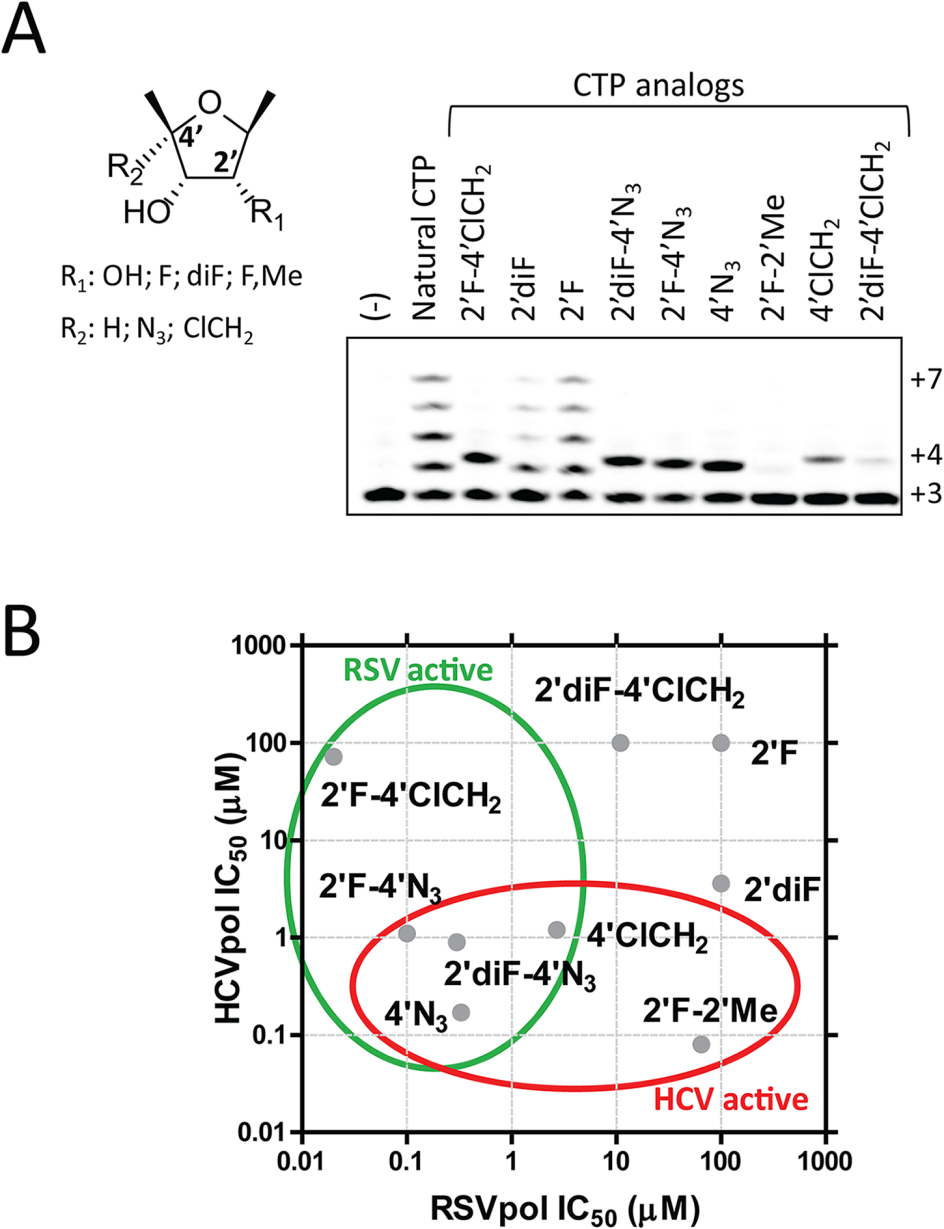
Identification of dual RSV/HCV polymerase inhibitors. **(A)** Incorporation by recombinant RSV polymerase of CTP analogs with a ribose containing either an OH, F, diF, or F-Me at the 2'-position, and either an H, N_3_, or ClCH_2_ at the 4'-position. Immediate chain terminators form a product at the +4 position, while natural substrate (CTP) fully extends the primer to the +7 position. (B) Comparative analysis of IC_50_ values of CTP analogs tested against RSV RNP complex versus HCV polymerase. Inhibitors of RSV polymerase are circled in green, and inhibitors of HCV polymerase are circled in red.

**Fig 6:**
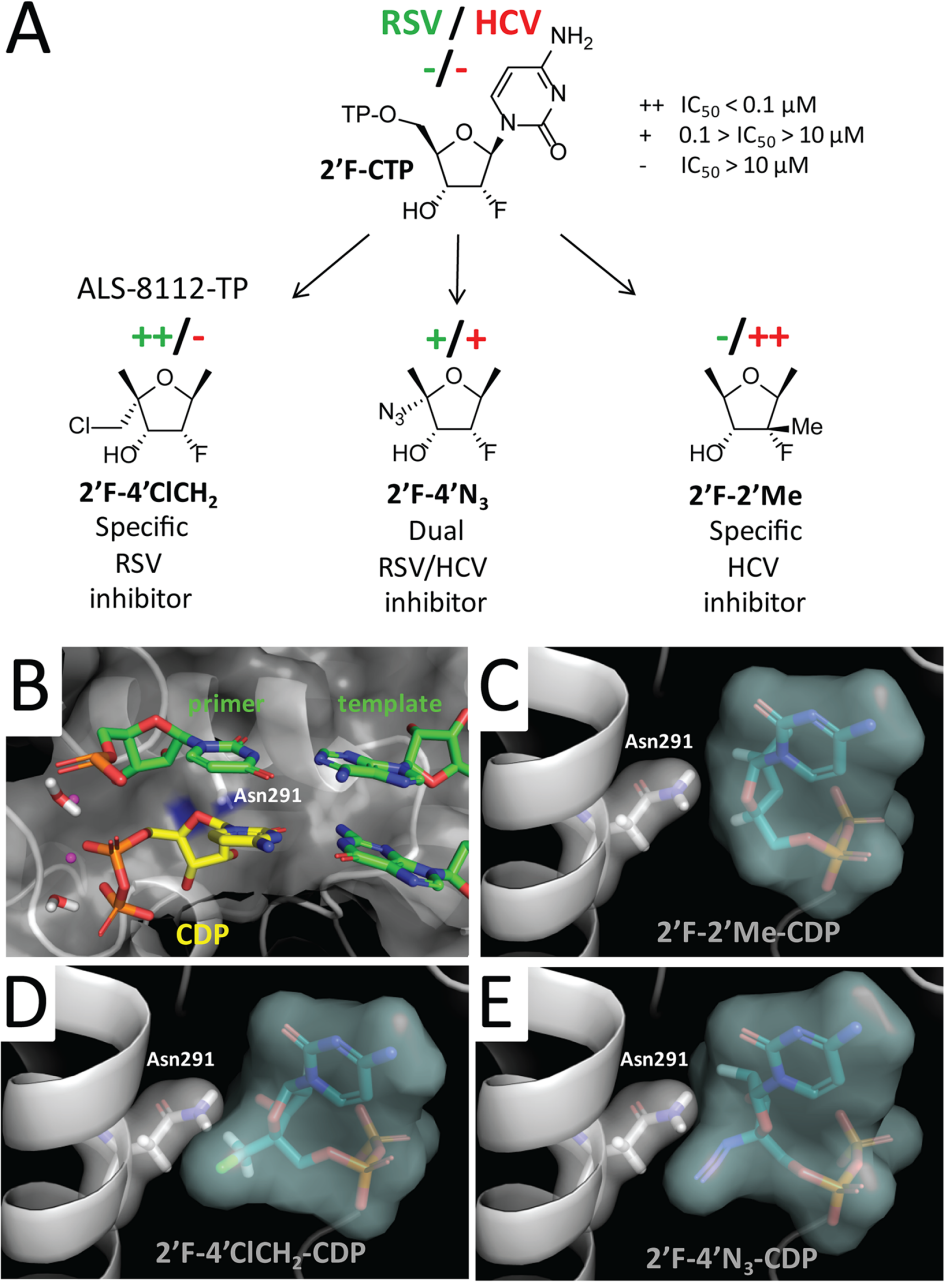
Rational design of ALS-8112 as a selective RSV inhibitor. **(A)** The nucleotide analog 2'-F-CTP is a substrate for both RSV and HCV polymerase, but it does not cause any inhibition by immediate chain termination. The addition of a 4'ClCH_2_ group (ALS-8112-TP) makes the molecule a selective inhibitor of RSV polymerase. The addition of a 2'Me group favors recognition by HCV polymerase, and the addition of a 4'N_3_ group causes dual RSV/HCV polymerase inhibition. **(B)** X-ray structure of natural CDP in the active site of HCV polymerase (PDB 4WTC, [28]). (**C, D**, and **E**) Docked binding modes of 2'F-2'Me-, 2'F-4'ClCH_2_-, and 2'F-4'N_3_-CDP, respectively.

To understand at the molecular level why ALS-8112-TP, unlike mericitabine-TP, is a substrate for RSV polymerase, we analyzed the incorporation profile of a series of structural intermediates between ALS-8112-TP and mericitabine-TP. Nine CTP analogs were modified to contain either an OH, F, diF, or F-Me at the 2'-position, and either an H, N_3_, or ClCH_2_ at the 4'-position (Fig 5A **)**. Some of these molecules included the triphosphate form of other clinically relevant molecules such as gemcitabine (2'diF-CTP) and balapiravir (4'N_3_-CTP). We found that 2'F- and 2'diF-CTP were substrates for RSV polymerase, but did not cause immediate chain termination (Fig 5A **)**. In contrast, 2'F-4'ClCH_2_- (ALS-8112-TP), 4'N_3_-, 2'F-4'N_3_- and 2'diF-4'N_3_-CTP were both substrates and chain terminators of recombinant RSV polymerase. Finally, 4'ClCH_2_-CTP seemed less efficiently recognized by the enzyme, while 2'diF-4'ClCH_2_- and 2'F-2'Me-CTP (mericitabine-TP) were almost completely inactive. Balapiravir-TP (4'N_3_-CTP) is a known inhibitor of HCV polymerase [26], which suggested that other CTP analogs could have dual RSV/HCV inhibition properties. This was further investigated by measuring the IC_50_ values of all compounds against HCV polymerase and RSV RNP complex. As expected, 2'F- and 2'diF-CTP did not efficiently inhibit either of the two polymerases, most likely because they did not cause immediate chain termination (Fig 5B) [27]. In contrast, 4'N_3_-, 2'F-4'N_3_-, 2'diF-4'N_3_-, and 4'ClCH_2_-CTP were all dual RSV/HCV polymerase inhibitors. These results were in agreement with the dual RSV/HCV antiviral effect of balapiravir in subgenomic replicons (Table 3). From this series of molecules, 2'F-4'ClCH_2_- (ALS-8112-TP) was the only CTP analog to efficiently inhibit RSV but not HCV polymerase. We conclude that, at the structural level, the selectivity of ALS-8112 towards RSV is provided by a combination of the 2'F- and the 4'ClCH_2_ moieties on the ribose group (Fig 6A and Fig H in S1 Text). On the other hand, the presence of the 2'Me-moiety led to a lack of recognition by RSV polymerase (Figs 5 and 6A).

To rationalize how the 2'F- and the 4'ClCH_2_ moieties contribute to the selectivity of ALS-8112-TP, we docked analogs of cytidine diphosphate (CDP) into the active site of the HCV polymerase ternary complex [28] (Fig 6B). Three binding metrics were computed for each docking pose: 1) RMSD (Å) to the natural CDP ligand; 2) number of hydrogen bonds formed with the crystallographically resolved water molecule anticipated to catalyze the formation of the new phosphodiester bond to the incoming nucleotide; and 3) number of hydrogen bonds formed with the RNA template (Fig IA in S1 Text). 2'F-2'Me-CDP had the lowest RMSD (Fig IA in S1 Text) and adopted a conformation comparable to CDP (Fig 6C). In comparison, 2'F-4'ClCH_2_-CDP (ALS-8112-DP) had the highest RMSD and the lowest number of hydrogen bonds (Fig IA in S1 Text). This unfavorable geometry was the result of a steric hindrance between the bulky 4'ClCH_2_ group and the side chain of Asn291 (Fig 6D and Fig IB in S1 Text), also placing a hydrophobic substitution in the proximity of a polar protein residue. In contrast, the more linear and charged 4'N_3_ group better occupied the binding pocket near the polar Asn291 (Fig 6E and Fig IB in S1 Text).

### Discrimination of the 4'ClCH_2_ moiety by RSV polymerase QUAD mutant

**Fig 7:**
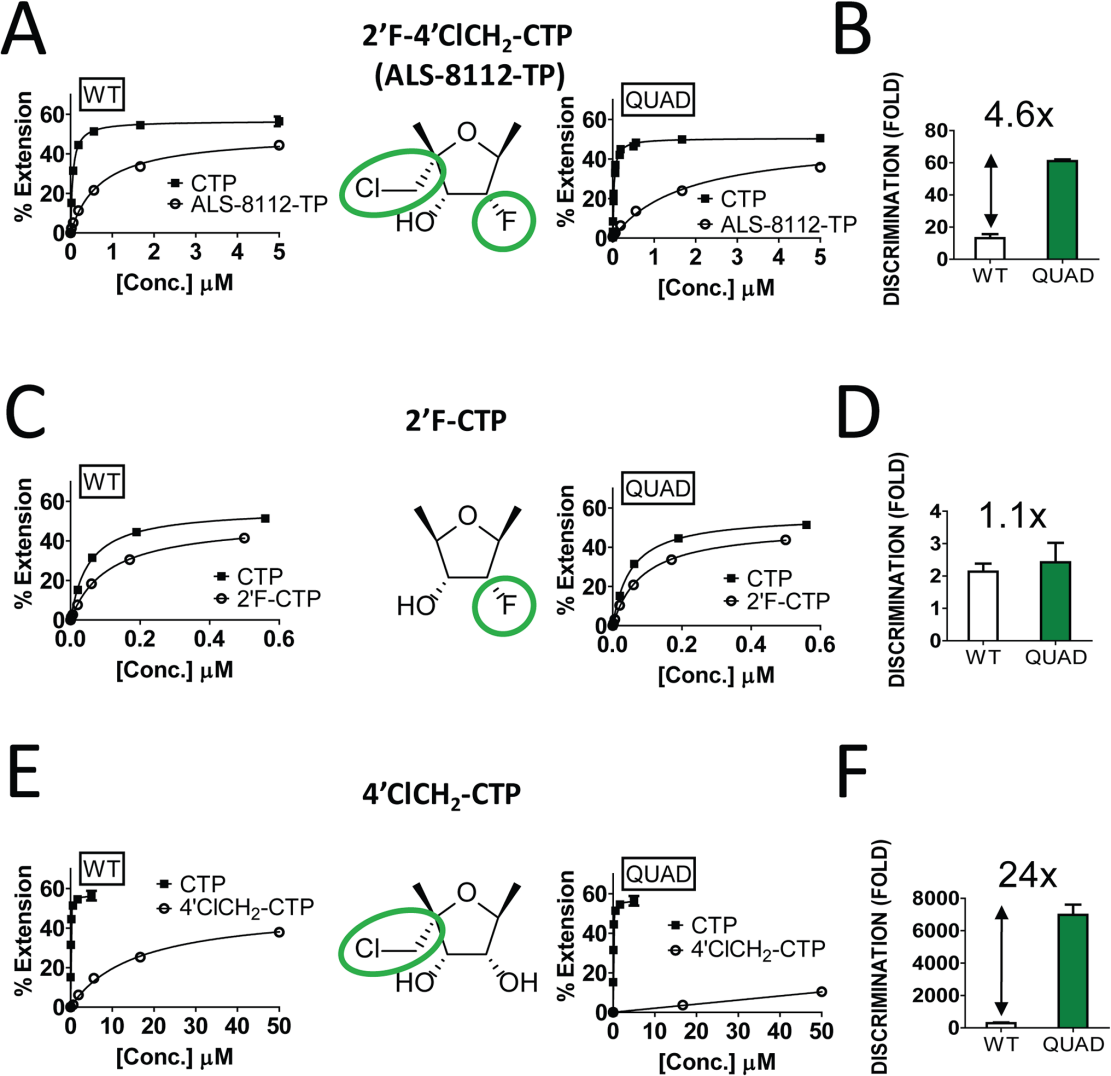
Contribution of the 4'ClCh_2_ group to the QUAD-mutant resistance to ALS-8112-TP. **(A)** The RSV L-P proteins (WT and QUAD) were incubated in the presence of GTP* + ATP and increasing concentrations of either CTP or ALS-8112-TP. Product formation was quantified and expressed as % primer extension from the +3 position (see calculation in Fig D in S1 Text). QUAD K_m CTP_ = 0.056±0.010 μM (*n* = 2), and QUAD K_m ALS-8112-TP_ = 1.74±0.34 μM (*n* = 2). K_m_ values for the WT enzyme were reported in Fig 4. **(B)** Fold discrimination for each enzyme was calculated as K_m CTP analog_ / K_m CTP_. **(C and D)** The RSV L-P proteins (WT and QUAD) were incubated in the presence of GTP* + ATP and increasing concentrations of 2'F-CTP. WT K_m 2'F-CTP_ = 0.12±0.014 μM (*n* = 2), and QUAD K_m 2'F-CTP_ = 0.07±0.017 μM (*n* = 2). **(E and F)** The RSV L-P proteins (WT and QUAD) were incubated in the presence of GTP* + ATP and increasing concentrations of 4'ClCH_2_-CTP. WT K_m 4'ClCH2-CTP_ = 16±2.7 μM (*n* = 2), and QUAD K_m 4'ClCH2-CTP_ = 198±18 μM (*n* = 2).

The four ALS-8112-selected amino acid mutations (QUAD: M628L, A789V, L795I, and I796V) were engineered into the RdRp region of the RSV L gene, and the recombinant L-P protein complex was produced. The wild-type enzyme and the QUAD mutant displayed a similar level of RdRp activity (Fig J in S1 Text). Using the primer extension assay described in Fig 4, the relative incorporation efficiencies between ALS-8112-TP substrate and natural CTP by RSV polymerase QUAD mutant led to a discrimination level of 61±1.1-fold (Fig 7A). Compared to the wild-type L-P enzyme (13±2.5-fold, Fig 4E), this represents a 4.6-fold increase in discrimination for ALS-8112-TP (Fig 7B). In order to understand at the molecular level which components of ALS-8112-TP contributed to the increased discrimination by the QUAD mutant, the same incorporation efficiency experiments were repeated with 2'F-CTP. Both WT RSV polymerase and the QUAD mutant efficiently recognized 2'F-CTP, which translated to low and comparable discrimination levels relative to CTP (Fig 7C and 7D). In contrast, 4'ClCH_2_-CTP was discriminated 288±48-fold by WT RSV polymerase, and 6,990±622-fold by the QUAD mutant (Fig 7E). This represents an overall resistance level of 24-fold (Fig 7F). Taken together, these results show that the increased discrimination of ALS-8112-TP by RSV polymerase QUAD mutant is conferred solely by the 4'ClCH_2_ moiety. Given the position of the last three of the four mutations within the RdRp domain, it is likely that motif B plays a critical role in the recognition by RSV polymerase of 4'-substitutions in nucleotide analogs.

## Discussion

In this study, we report the first detailed molecular characterization of ALS-8176, a ribonucleoside analog that inhibits RSV replication in vitro and is efficacious in RSV-infected adult volunteers [18,19]. We provide data demonstrating that ALS-8176 was efficacious in RSV-infected African Green monkeys (Fig 1E), and that the parent nucleoside, ALS-8112, is a broad-spectrum yet selective inhibitor of RNA viruses within the *Paramyxoviridae* family, which includes several other human pathogens such as the parainfluenza viruses (Table 1, Fig 1B, and Fig D in S1 Text). ALS-8112 also inhibited the replication of vesicular stomatitis virus, a rhabdovirus. However, ALS-8112 did not inhibit RNA viruses distant from the *Paramyxoviridae* family, such as influenza (segmented ssRNA-) or HCV (ssRNA+) (Table 1). This antiviral profile is consistent with the mode of action of ALS-8112 specifically targeting the RdRp domain of the viral L protein, which is conserved within the Mononegavirales [29–31]. In viruses from this order, the L protein mediates all the enzymatic functions required for genomic replication and transcription, including the RdRp activity. Our mechanistic studies provide several independent lines of evidence that ALS-8112 specifically targets the RdRp function of RSV polymerase. Viruses continuously passaged in the presence of ALS-8112 in vitro eventually developed drug resistance-associated mutations (Fig 2A). Three of the four amino acid changes associated with ALS-8112 resistance are positioned within motif B, in the vicinity of the CRIII region that contains the conserved motif C residues _810-_GDNQ_-813_ responsible for nucleotide incorporation by paramyxovirus RNA polymerases [32,33]. These mutations were introduced into the RSV L gene to measure the viral polymerase activity with a cell-based minigenome assay. In this assay, the presence of the four mutations in the RSV polymerase gene was associated with a 39-fold loss in antiviral potency, without any significant change in raw luciferase signal (Fig 2B, Fig CA and CB in S1 Text).

In order to demonstrate that the inhibition of RSV replication by ALS-8112 is mediated by its 5'-triphosphate metabolite, we synthesized the 5'-iodo derivative of ALS-8112 (ALS-8112-I) as a negative control. As expected, ALS-8112-I was inactive against RSV (Fig A in S1 Text). The mechanism of action of ALS-8112 involving its 5'-triphosphate as the active form was further supported by the convergence in antiviral spectrum from the enzymatic assays directly showing that ALS-8112-TP inhibits the RNA polymerase of RSV and PIV-1 (Fig 2C and Table 2), but not those of influenza or HCV (Table 2, Fig E in S1 Text). Other direct evidence for the mechanism of action of ALS-8112 is provided by single nucleotide incorporation experiments using purified RSV polymerase expressed as a recombinant L-P dimer (Fig 4A). In this assay, the RSV L-P protein specifically incorporated ALS-8112-MP to the growing RNA chain opposite a guanine on the template (Fig 4B and 4C). The result of ALS-8112-MP incorporation was immediate and complete chain termination of RNA synthesis. This is the hallmark of many potent nucleoside/nucleotide analogs that have been used against other viruses such as HIV, HBV, and HCV (reviewed in [12,34,35]). For RSV and other paramyxoviruses, ALS-8112 is the first example of an antiviral nucleoside analog that specifically targets the viral polymerase and causes chain termination. From an enzymatic standpoint, ALS-8112-TP can be considered an efficient substrate for nucleotide incorporation by RSV polymerase, with only a 13-fold discrimination relative to natural CTP (Fig 4D and 4E). In the presence of the QUAD mutations, the level of ALS-8112-TP discrimination was 61-fold (Fig 7A). This represents a 4.6-fold increase compared to the wild-type enzyme (Fig 7B). This is significantly lower than the resistance phenotype measured in the RSV minigenome and in the crude RNP assays, both monitoring full genomic transcription events. Our interpretation is that the 4.6-fold increase in discrimination at the single nucleotide level might be amplified with the total number of cytidine incorporation possibilities occurring during complete RSV genome replication and transcription. Importantly, we found with the recombinant RSV polymerase that the resistance phenotype provided by the QUAD mutations was solely caused by the 4'ClCH_2_ moiety (Fig 7C–7F). Given the position of the last three of the four mutations within the RdRp domain (Fig 2A), it is likely that motif B plays a critical role in the recognition by RSV polymerase of 4'-substitutions in nucleotide analogs. Further experiments will be needed to fully understand the individual contribution of each of the QUAD mutations towards the resistance of RSV polymerase against ALS-8112.

Another aim of this study consisted in evaluating at the molecular level the effect of structural changes in ALS-8112-TP on the recognition by RSV polymerase. We found that the triphosphate form of the ribonucleoside analog mericitabine (2'F-2'Me-CTP) once developed for the treatment of HCV infection is > 5,000-fold less efficiently incorporated than its natural CTP counterpart (Fig 4D and 4E). Interestingly, the recombinant RSV polymerase complex incorporated other cytidine analogs known to inhibit HCV polymerase (Fig 5A), such as balapiravir-TP (4'N_3_-CTP) [26]. This led us to investigate further the effect of CTP ribose modifications on the dual inhibition of RSV and HCV polymerase. From a series of nine CTP analogs, four were able to inhibit both RSV and HCV polymerase (Fig 5B). The only nucleotide selective towards RSV polymerase was ALS-8112-TP (2'F-4'ClCH_2_-CTP). From these experiments, we conclude that the poor enzymatic recognition of ALS-8112-TP by HCV RNA polymerase is mediated by a combination of the 2'F and the 4'ClCH_2_ substitutions (Fig 6A and Fig H in S1 Text). Recently, Appleby *et al*. determined the crystal structure of the HCV RNA polymerase in ternary complex with an incoming nucleotide diphosphate (DP) [28]. Computer modeling of CDP analogs inside the active site of HCV polymerase revealed that the 4'ClCH_2_ group of ALS-8112-DP interacts unfavorably with Asn291, whereas a 4'N_3_ substitution is better tolerated (Fig 6B–6E and Fig I in S1 Text), in agreement with the enzyme inhibition data. The amino acid Asn291 is part of motif B, which is well conserved within plus-strand RNA virus polymerases [36]. For instance, in the crystal structure of poliovirus RNA polymerase, the equivalent Asn297 adopts a similar orientation towards the incoming nucleotide [37], and its mutation causes significant changes in the rate of catalysis [38]. However, that part of the sequence varies in paramyxovirus and other negative-strand RNA virus polymerases [30,39]. Although our resistance-mutation selection study also suggests that residues in motif B may also play a role in the recognition of ALS-8112-TP by RSV polymerase, the structural basis for this observation is unknown. The rational design of RSV polymerase inhibitors is limited by the lack of a high resolution crystal structure for any paramyxovirus polymerase. Despite this limitation, the combination of molecular modeling and enzymatic analysis such as the one presented in this study should help to further optimize inhibitor potency and selectivity.

Can advanced antiviral nucleosides be repurposed against neglected or emerging viral infections? The HCV inhibitor, sofosbuvir, is the only FDA-approved ribonucleotide to specifically inhibit a viral RNA polymerase, but it is inactive against viruses distant from HCV [13]. The molecular reason why anti-HCV drugs like sofosbuvir or mericitabine are not active against negative-strand RNA viruses is an important question that has not been addressed. In this respect, we provide the first mechanistic study to measure the selectivity of clinically-relevant nucleotides by comparing their inhibition profile against the polymerase of a positive- (HCV) and a negative-strand (RSV) RNA virus. We show that minor structural modifications on the ribose of nucleotides can dramatically change their selectivity (Figs 5 and 6). From this work, further experiments will be needed to understand the true potential of ALS-8112/ALS-8176 against other non-segmented negative-strand RNA viruses related to RSV, such as filoviruses. In particular, it will be important to pinpoint the precise molecular interaction between ALS-8112-TP and the polymerase of viruses other than RSV. From a mechanistic standpoint, the study presented here is the first comparative analysis using recombinant RSV and HCV polymerases to elucidate the mechanism of action and selectivity of nucleotide analogs. Ultimately, understanding nucleotide selectivity towards distant viral RNA polymerases could not only be used to repurpose existing antivirals against previously unaddressed viral infections, but also to design novel molecules.

## Materials and Methods

### Compounds

ALS-8176 (also referred to as ALS-008176), ALS-8112, ALS-8112-MP, ALS-8112-TP, and all derivatives of ALS-8112-TP were synthesized at Alios BioPharma (South San Francisco, CA, USA) according to described procedures [18]. ALS-8112-I was synthesized as described in S1 text. The parent nucleoside ALS-8112 and its prodrug ALS-8176 were stored at 4°C in dimethyl sulfoxide (DMSO), and all the phosphorylated species were reconstituted in water, aliquoted, and stored at -80°C.

### Viruses and cell culture

RSV stocks were purchased from American Type Culture Collection (ATCC, Cat. #VR-1540 for RSV A2; VR-26 for RSV Long, and VR-1400 for RSV B1). Recombinant RSV expressing the far red fluorescent protein mKate2 was previously described [40]. The viruses were amplified in Vero cells and titrated in Vero cells by the amount of virus suspension producing infection in 50% of cell cultures inoculated (TCID_50_). The A549 cells (ATCC) were maintained in Ham's F12 media with 10% fetal bovine serum [FBS]) and 1% penicillin-streptomycin and HEp2 cells were maintained in Dulbecco's Modified Eagle medium (DMEM)/F-12 with 5% FBS and 1% penicillin-streptomycin. Measurement of in vitro intracellular NTP formation was performed as described in S1 text.

### Measurement of RSV inhibition by RT-PCR

Determination of the EC_50_ and CC_50_values of ALS-8112 in the RSV assays was performed by the following procedure. On the first day, 20,000 HEp-2 cells per well were plated in a 96-well plate. Each compound was serially diluted (1:3) up to 9 distinct concentrations. For the three-dimensional tissue culture system, primary NHBE cells (EpiAirway PC-12, MatTek Corporation) were provided in columns embedded in agar plates [20]. On the day of their arrival, 1.5 mL of warm media (AIR-100-ASY) was added to each well of 6-well plates and then the columns were placed into the 6-well plates and incubated overnight in a 37°C humidified, 5% CO_2_ atmosphere. Cells were pre-incubated with compounds for 24 hours at 37°C in a 5% CO_2_ atmosphere. After 24 hours of pre-incubation with compounds, RSV A2, Long, or B1 at a multiplicity of infection (MOI) of 0.5 was added to the cells, except for the background controls. The plate was then incubated for additional 4 days in the same conditions and at the end of the incubation 50 μL the supernatant from each well of the plate was collected. The RSV viral RNA was isolated from the collected supernatant of each well using a MagMAX Viral RNA Isolation Kit (Life Technologies) automated through the MagMAX Express-96 Deep Well Magnetic Particle Processor (Life Technologies). The viral RNA was then quantified by Real Time polymerase chain reaction (RT-PCR) using the primer/probe set (see sequences in S1 text).

### RSV subgenomic replicon assay

The RSV subgenomic replicon (Apath, Brooklyn, NY, USA) was used as previously described [41]. The HeLa-derived cells containing the stable RSV replicon were cultured in DMEM containing 4500 mg/L D-glucose, L-glutamine, and 110 mg/L sodium pyruvate. The medium was further supplemented with 10% (v/v) FBS (Mediatech), 1% (v/v) penicillin/streptomycin (Mediatech), and 10 μg/mL of Blasticidin (BSD) (Invivogen). Cells were maintained at 37°C in a humidified 5% CO_2_ atmosphere. On the first day, 5000 RSV replicon cells per well were plated in a 96-well plate. On the following day, compounds to be tested were solubilized in 100% DMSO to 100 × the desired final testing concentration. Cells were incubated with compounds for 7 days at 37°C in a 5% CO_2_ atmosphere before measurement of the luciferase readout. Cell viability (CC_50_) was measured with a CellTiter-Glo cell proliferation assay (Promega).

### Selection of ALS-8112 resistance mutations

RSV A2 virus was passaged in increasing concentrations of ALS-8112 in duplicate, together with a parallel passage with media containing no drug as a control. After each passage, viruses from each pool were harvested, titrated by TCID_50_, and stored for the next passage and further characterization. A preliminary phenotypic assessment of both treated pools after > 35 passages at a point when the ALS-8112 concentration had reached 16 μM indicated a loss in antiviral potency for ALS-8112 in the treated virus pool vs. the control virus pool. This was used as the basis to trigger RSV genome sequencing of all virus pools.

### African Green monkey efficacy model

Eight RSV-seronegative African Green monkeys (*Chlorocebus sabaeus*) approximately 4–8 years of age were obtained from PrimGen (Hines, IL). Animals were housed at BioQual Inc. (Rockville, MD) under BSL2 conditions as specified by the Association for Assessment and Accreditation for Laboratory Animal Care (AAALAC) guidelines. All protocols were IACUC approved. The weight range of the animals varied from ~3.7–8.0 kg. Four of the animals were male. Animals were acclimated to the general housing conditions for at least 6 weeks before the study start. Prior to dose administration, sample collections or viral challenge, animals were anesthetized with ketamine hydrochloride. All ALS-8176 dose formulations were prepared fresh on each day of dosing. The dose formulation was administered via oral gavage according to standard procedures. During the ALS-8176 dosing period, animals were fasted overnight and all ALS-8176 doses were given in the fasted state. During the dosing days, animals were fed at least 1 hour after the morning dose and no later than 2 hours prior to the evening dose. ALS-8176 and vehicle were administered 24 hours prior to the viral challenge (Day -1).

### RSV minigenome assay

A modified RSV minigenome assay based on a previously described method [40,42] was used to assess the phenotypic shift in the quadruple mutation. In brief, HEp-2 cells were plated in 6-well plates at the density of 0.5 million cells/well. ALS-8112 was serially diluted and added to each of the wells and further incubated over night. On the next day, modified vaccinia virus Ankara-T7 (MVA-T7) at the MOI of 1 was added to provide T7 RNA polymerase [43]. After 2 hours of viral transduction, each well was transfected with Fugene 6 (Promega) with 1.25 μg mixture of 6 plasmids including minigenome (pGem.RSV.M5.Luc), plasmids encoding human codon bias-optimized N, P, M2-1, L genes as well as control plasmid encoding renilla luciferase pRL-SV40. After 48 hours of further incubation, the firefly luciferase as well as renilla luciferase signals from each well were measured with dual-luciferase reporter assay system (Promega). The normalized signals (firefly luciferase over renilla luciferase) were plotted over ALS-8112 concentrations to obtain IC_50_ values.

### RSV RNP complex and other viral RNA polymerase assays

Cell fractionation and extraction of crude RSV and PIV-1 RNP complexes were performed as described [23]. Unless otherwise specified, nucleoside triphosphate (NTP) concentrations were around their K_m_ value: 0.1 μM CTP, 1 μM ATP, 2 μM UTP, and 500 μM GTP. The radioactive tracer was either 10 μCi [α-^33^P]rCTP (CTP competitive mode) or 15 μCi [α-^33^P]rGTP (non-CTP competitive mode) together with 0.1 μM GTP. The NTP concentration for the competition assay was either 1 or 100 μM for CTP, ATP, GTP, and UTP. The reaction was initiated with the addition of 1.5 μL of the virus-infected cell extract and incubated for 2 hours at 30°C in a final volume of 30 μL. Unless otherwise specified, the standard reaction was stopped by adding 25 μL of Tris-sodium-EDTA buffer containing 10 mM Tris-HCl pH 8, 150 mM NaCl, and 100 mM EDTA. Processing of samples included elution through a G-50 spin column (GE Healthcare), phenol-chloroform extraction, and ethanol precipitation. Air-dried RNA samples were reconstituted in 20 μL TBE urea gel loading buffer (Invitrogen), and migrated through a 6% TBE urea PAGE gel for 1 hour at 190 V. The gel was dried and exposed to a phosphor-screen that was scanned with a Storm 860 phosphorImager (Molecular Dynamics). Biochemical assays for the inhibition of influenza and HCV polymerases were performed as previously described [17,27].

### Single nucleotide incorporation by recombinant RSV L-P dimer

The recombinant RSV polymerase complex was produced by co-expressing the L and P proteins of RSV in insect cells as previously described [25]. Unless otherwise specified, RNA polymerase reaction samples consisted of 0.2 μM of an oligonucleotide template sequence derived from the RSV leader promoter (5'-UUUGUUCGCGU-3') and 0.2 μM recombinant RSVL-P polymerase together with 200 μM 5'-pACGC primer, mixed in a buffer containing 20 mM Tris pH 7.5, 10 mM KCl, 2 mM dithiothreitol, 0.5% triton, 10% DMSO, 0.2 U/μL RNasin (Ambion), and 6 mM MgCl_2_. Reactions were started at 30°C by adding specific NTPs in a final volume of 10 μL. The radioisotope tracer used for this assay was α^33^P-GTP. Reactions were stopped after 30 minutes by adding an equal volume of gel loading buffer (Ambion). Samples were denatured at 95°C for 5 minutes, and run for 1.5 hours at 80 W in a 22.5% polyacrylamide urea sequencing gel. After the gel was dried, the product of migration was exposed to a phosphor-screen and scanned as previously described.

### Molecular modeling

All calculations were carried out using the Schrӧdinger software suite (Schrӧdinger, LLC). A molecular model of the HCV NS5B polymerase elongation complex (PDB 4WTC) [28], was generated. The protein structure was prepared with Protein Preparation Wizard. Docking grids were computed with Glide. To ensure adequate sampling of metal-chelating conformations of the diphosphate moiety during docking, ligand input conformation ensembles were generated using MacroModel, with torsional constraints in place for C4’, C5’ and the diphosphate moiety, as observed for CDP in 4WTC. Ligand binding to the HCV NS5B polymerase catalytic site was evaluated with Glide docking calculations: SP precision level, with reference core constraints based on the CDP X-ray binding mode revealed in 4WTC (core atoms encompassing the diphosphate moiety, C5’, C4’, ribose O, C1’, and heavy atoms of the nucleobase). Up to 20 binding modes were collected for each ligand, without post-docking minimization.

### Data analysis and statistics

Sigmoidal dose-response curves used to generate 50% or 90% inhibitory or effective concentrations were analyzed by nonlinear regression using the four-parameter logisitic equation (GraphPad Prism). All data are presented as mean ± SEM or SD as specified, with experimental uncertainties identified by error bars. Statistical significance was calculated using a 2-tailed Student's *t* test. Differences with a *P* value of less than 0.05 were considered statistically significant.

## Supporting Information

S1 Text Supporting Information tables, figures, and text. **Chemical synthesis of ALS-8112-I.** Chemical synthesis steps leading to the formation of 4’-Chloromethyl-2’,5’-dideoxy-2’-fluoro-5’-iodocytidine. **Table A. Inhibition potency of ALS-8112 against RSV A and B clinical isolates.** Each clinical isolate was grown in HEp-2 cells, and the EC_50_/EC_90_ values were determined as described for RSV Long and A2 strains. **Table B. Primer sequences used for RT-PCR detection of RSV RNA levels.** For each strain of RSV used, a specific set of primers was designed in order to maximize the efficiency. **Fig A. Inhibition of RSV subgenomic replicon by ALS-8112-I.** (**A**) Chemical structure of 2'-fluoro-4'-chloromethyl-5'-iodo cytidine, or ALS-8112-I. (**B**) In vitro inhibition potency of ALS-8112 and ALS-8112-I against the RSV replicon. **Fig B. Inhibition by ALS-8112 of RSV replication in normal, human-derived tracheal/bronchial epithelial cells**. ALS-8112 dose-response curve of viral RNA, as measured by qRT-PCR. The antiviral activity of ALS-8112 was measured in cells from three individual human donors ranging from 5 months to 23 years of age. **Fig C. Effect of the QUAD mutations on the inhibition potency of ALS-8112 and ALS-8112-TP.** (A) Representative ALS-8112 dose-response curve showing the antiviral activity of ALS-8112 in the RSV minigenome assay expressing either the wild-type (WT) or the QUAD-mutant L protein. (B) Raw firefly luciferase activity of WT and QUAD-mutant minigenome. (C) Representative urea PAGE analysis of cell-free WT RSV-dependent RNA transcript in the presence of increasing concentrations of ALS-8112-TP. (D) Representative urea PAGE analysis of cell-free QUAD-mutant RSV-dependent RNA transcript in the presence of increasing concentrations of ALS-8112-TP. (E) ALS-8112 dose-response quantitation of changes in RNA transcripts (% inhibition) for WT and QUAD-mutant RSV RNA complex activity. **Fig D. Inhibition potency of ALS-8112 against RSV and other RNA viruses.** A representative ALS-8112 EC_50_ and CC_50_ curve is reported for each virus. The 50% cutoff (dotted line) was used as basis to determine the CC_50_ value > 100 μM for each assay. **Fig E. Inhibition potency of ALS 8112-TP against RSV and other viral RNA polymerases.** The HCV polymerase inhibition assay was performed in 96-well plate as previously reported [1]. The PIV-1 polymerase gel-based assay was identical to the RSV polymerase assay using crude RNP cell extract. The IAV polymerase gel-based assay was performed as previously described [2] with a single 100 μM concentration of ALS-8112-TP, which was used for the basis of the IC_50_ > 100 μM. **Fig F. Selectivity of CTP analogs towards human mitochondrial RNA polymerases.** The DdRp assay with human mitochondrial RNA polymerase was performed under single turnover conditions where enzyme concentration is in excess of the primer/template [3]. Therefore, the ^33^P-RNA/DNA primer / template was used at a concentration of 100 nM, together with 320 nM enzyme, in the presence of 100 μM of each NTP, 10 mM MgCl_2_, 50 mM NaCl, 40 mM Tris, pH 7.5, and 1 mM DTT. CTP was used as positive control for single nucleotide incorporation, and UTP/ATP/GTP were controls of mis-incorporation. **Fig G. Efficiency of incorporation of mericitabine-TP by RSV polymerase.** The RSV L-P complex was incubated in the presence of GTP + ATP and increasing concentrations of mericitabine-TP: lane 0, 0 μM, lane 1, 1.2 μM; lane 2, 3.7 μM; lane 3, 11 μM; lane 4, 33 μM, lane 5, 100 μM; lane 6, 300 μM. Product formation of mericitabine-TP incorporation was quantified and expressed as % primer extension from the +3 position, calculated as follows: % primer extension = P4 / (P3+P4) x 100. **Fig H. Exploration of the SAR around RSV vs. HCV polymerase inhibition.** (A) ALS-8112-TP, a selective inhibitor of RSV polymerase, contains a 2'F and a 4'ClCH_2_ group. Other substitutions at the 2'- and 4'-position were explored. (B) Within the 2'OH series, both 4'N_3_-CTP and 4'ClCH_2_-CTP inhibited RSV and HCV polymerase. Within the 2'difluoro series, 2'diF-4'N_3_-CTP inhibited RSV and HCV polymerase, while 2'diF-4'ClCH_2_-CTP inhibited neither of them. **Fig I. Molecular modeling of ALS-8112-DP into HCV polymerase.** (A) Calculated binding metrics for nucleotide analogs. Three binding metrics were computed for each docking pose: 1) RMSD (Å) to the natural CDP ligand; 2) number of hydrogen bonds formed with the crystallographically resolved water molecule anticipated to catalyze the formation of the new phosphodiester bond to the incoming nucleotide; and 3) number of hydrogen bonds formed with the RNA template (B) Docked binding modes for 2'F-2'Me-, 2'F-4'ClCH_2_-, and 2'F-4'N_3_-CDP were overlaid with the CDP (yellow) X-ray binding mode for reference. **Fig J. Comparison of RdRp activity between WT and QUAD RSV L-P polymerase complex.** The assay was performed as described in Fig 4. The 11-mer template was used in combination with radiolabled GTP (G*) and ATP, so that the primer could be extended by three bases (+3). The intensity of the +3 product was used to compare the activity of the WT and QUAD mutant proteins.
(PDF)
