# Supplementary material for: Molecular Basis for the Selective Inhibition of Respiratory Syncytial Virus RNA Polymerase by 2'-Fluoro-4'-Chloromethyl-Cytidine Triphosphate
Source: PLoS Pathog. 2015 Jun 22;11(6):e1004995. doi: 10.1371/journal.ppat.1004995 (PMC4476725; doi:10.1371/journal.ppat.1004995)
Supplement: S1 Text — Chemical synthesis of ALS-8112-I. Chemical synthesis steps leading to the formation of 4’-Chloromethyl-2’,5’-dideoxy-2’-fluoro-5’-iodocytidine. Table A. Inhibition potency of ALS-8112 against RSV A and B clinical isolates. Each clinical isolate was grown in HEp-2 cells, and the EC50/EC90 values were determined as described for RSV Long and A2 strains. Table B. Primer sequences used for RT-PCR detection of RSV RNA levels. For each strain of RSV used, a specific set of primers was designed in order to maximize the efficiency. Fig A. Inhibition of RSV subgenomic replicon by ALS-8112-I. (A) Chemical structure of 2'-fluoro-4'-chloromethyl-5'-iodo cytidine, or ALS-8112-I. (B) In vitro inhibition potency of ALS-8112 and ALS-8112-I against the RSV replicon. Fig B. Inhibition by ALS-8112 of RSV replication in normal, human-derived tracheal/bronchial epithelial cells. ALS-8112 dose-response curve of viral RNA, as measured by qRT-PCR. The antiviral activity of ALS-8112 was measured in cells from three individual human donors ranging from 5 months to 23 years of age. Fig C. Effect of the QUAD mutations on the inhibition potency of ALS-8112 and ALS-8112-TP. (A) Representative ALS-8112 dose-response curve showing the antiviral activity of ALS-8112 in the RSV minigenome assay expressing either the wild-type (WT) or the QUAD-mutant L protein. (B) Raw firefly luciferase activity of WT and QUAD-mutant minigenome. (C) Representative urea PAGE analysis of cell-free WT RSV-dependent RNA transcript in the presence of increasing concentrations of ALS-8112-TP. (D) Representative urea PAGE analysis of cell-free QUAD-mutant RSV-dependent RNA transcript in the presence of increasing concentrations of ALS-8112-TP. (E) ALS-8112 dose-response quantitation of changes in RNA transcripts (% inhibition) for WT and QUAD-mutant RSV RNA complex activity. Fig D. Inhibition potency of ALS-8112 against RSV and other RNA viruses. A representative ALS-8112 EC50 and CC50 curve is reported for each virus. The [file ppat.1004995.s001.pdf]

S1 Text - Chemical synthesis of ALS-8112-I

4'-Chloromethyl-2',5'-dideoxy-2'-fluoro-5'-iodocytidine (**2**)

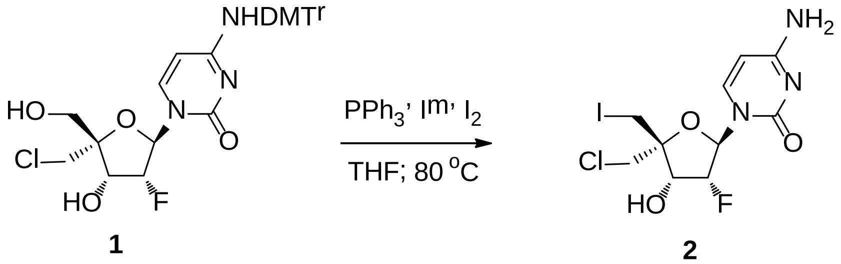

A mixture of **1** (prepared according to G. Wang et al., J. Med. Chem. 2015, accepted for publication) (80 mg, 0.13 mmol), Ph_3P (140 mg, 0.53 mmol), imidazole (36 mg, 0.53 mmol), and iodine (68 mg, 0.27 mmol), in THF (2.0 mL) was stirred at 80 ^{o}C for 16 h and then cooled to rt. The mixture was diluted with EtOAc and washed with 7% aq. $Na_2S_2O_3$, brine and dried (Na_2SO_4). Chromatography on silica gel with 4-20% MeOH in CH_2Cl_2 followed by RP-HPLC purification (0-60% B; A: 50 mM aq. TEAA, B: 50mM TEAA in MeCN) yielded **2** as a white solid (22 mg, 42%).

^{1}H NMR (DMSO-d_6) δ 7.61 (d, J = 6.7 Hz, 1H, H-6), 7.36, 7.29 (2 s, 2H, NH_2), 6.01 (d, J = 14.1 Hz, 4.9 Hz, 1H, H-1'), 5.74 (d, J = 5.9 Hz, 1H, H-5), 5.38 (d, J = 53.2 Hz, 1H, H-2'), 4.48 (br s, 1H, H-3'), 3.95 (d, J = 11.7 Hz, 1H, H-5'a), 3.75 (d, J = 11.7 Hz, 1H, H-5'a), 3.57 (m, 2H, CH_2Cl). ^{19}F NMR (DMSO-d_6) δ -202.85 (m). MS, m/e 403.9 $(M + 1)^+$.

S1 Text - Materials and Methods

Panel of in vitro antiviral assays. The PIV-3 antiviral assay was performed in A549 cells infected with the human PIV-3 C243 strain at a MOI of 0.1, for a total duration of 7 days. EC_{50} values were determined based on the quantitation on viral RNA by qRT-PCR. The HCV antiviral assay was conducted in the standard Huh-7 replicon, as previously described [4]. The RSV replicon assay was also performed as previously described [5]. The human rhinovirus assay was performed in HeLa-OHIO cells infected with 70 $TCID_{50}$ of HRV-1b. After 4-6 days, virus-induced cell death (CPE) was measured using CellTiter Glo Luminescent Cell Viability Assay (Promega). The VSV assay was performed in A549 cells, and CPE was measured using CellTiter Glo reagent 48h after infection. For the influenza assay, A549 cells were infected with 250 IU/well of influenza strain A/WSN/33 (H1N1) incubated for 20h. The cell culture supernatant was discarded and replaced with 25 µM 2'-(4-methylumbelliferyl)-a-D-N-acetylneuraminic acid (Sigma-Aldrich) dissolved in 33 mM MES pH 6.5. Fluorescence emitted from the cleaved neuraminidase substrate was measured with excitation and emission filters of 355 and 460 nm, respectively, on a Victor X3 multi-label plate reader (Perkin Elmer, Waltham, MA). For each cell line, cytotoxicity (CC_{50}) was measured in uninfected cells at the same range of compound concentration using CellTiter Glo Luminescent Cell Viability Assay. Changes in cell viability across assays and cell lines may be explained by differences in assay duration and/or differences in compound metabolism depending on the cells.

References

1. Fung A, Jin Z, Dyatkina N, Wang G, Beigelman L, et al. (2014) Efficiency of Incorporation and Chain Termination Determines the Inhibition Potency of 2'-Modified Nucleotide Analogs against Hepatitis C Virus Polymerase. Antimicrob Agents Chemother 58: 3636-3645.
2. Jin Z, Smith LK, Rajwanshi VK, Kim B, Deval J (2013) The ambiguous base-pairing and high substrate efficiency of T-705 (Favipiravir) Ribofuranosyl 5'-triphosphate towards influenza A virus polymerase. PLoS One 8: e68347.
3. Arnold JJ, Sharma SD, Feng JY, Ray AS, Smidansky ED, et al. (2012) Sensitivity of mitochondrial transcription and resistance of RNA polymerase II dependent nuclear transcription to antiviral ribonucleosides. PLoS Pathog 8: e1003030.
4. Klumpp K, Leveque V, Le Pogam S, Ma H, Jiang WR, et al. (2006) The novel nucleoside analog R1479 (4'-azidocytidine) is a potent inhibitor of NS5B-dependent RNA synthesis and hepatitis C virus replication in cell culture. J Biol Chem 281: 3793-3799.
5. Wang G, Deval J, Hong J, Dyatkina N, Prhavc M, et al. (2015) Discovery of 4'-chloromethyl-2'-deoxy-3',5'-di-O-isobutyryl-2'-fluorocytidine (ALS-8176), a first-in-class RSV polymerase inhibitor for treatment of human respiratory syncytial virus infection. J Med Chem 58: 1862-1878.

Table A

Inhibition potency of ALS-8112 against RSV A and B clinical isolates

Isolate	Subtype	ALS-8112 EC_{50} (µM)	ALS-8112 EC_{90} (µM)
18537	RSV B	0.21 ± 0.06	0.99 ± 0.22
9320	RSV B	0.09 ± 0.03	0.96 ± 0.16
1144	RSV B	0.29 ± 0.02	1.47 ± 0.22
1262	RSV B	0.11 ± 0.03	2.18 ± 0.27
Pep 121	RSV B	0.32 ± 0.07	2.07 ± 0.43
A1997-12-35	RSV A	0.18 ± 0.04	2.46 ± 0.15
A1998-03-02	RSV A	0.26 ± 0.06	2.15 ± 0.27
A1998-12-21	RSV A	0.09 ± 0.02	1.11 ± 0.54
A2000-03-04	RSV A	0.18 ± 0.02	2.18 ± 0.24
A2001-02-20	RSV A	0.1 ± 0.01	1.1 ± 0.53
A2001-03-12	RSV A	0.45 ± 0.07	2.33 ± 0.35

Table B

Primer sequences used for RT-PCR detection of RSV RNA levels

Strain	Upstream Primer	Probe	Downstream Primer
RSV A2	5’GCT CTT AGC AAA GTC AAG TTG AAT GA3’	6FAM-ACA CTC AAC AAA GAT CAA CTT CTG TCA TCC AGC-MGBNFQ	5’TGC TCC GTT GGA TGG TGT ATT3’
RSV Long	5’GCT CTT AGC AAA GTC AAG TTG AAT GA3’	6FAM-ACA CTC AAC AAA GAT CAA CTT CTG TCA TCT AGC-MGBNFQ	5’TGC TCC GTT GGA TGG TGT ATT3’
RSV B1	5’GAT GGC TCT TAG CAA AGT CAA GTT AA3’	6FAM-TGA TAC ATT AAA TAA GGA TCA GCT GCT GTC ATC CA-MGBNFQ	5’TGT CAA TAT TAT CTC CTG TAC TAC GTT GAA3’

Figure A

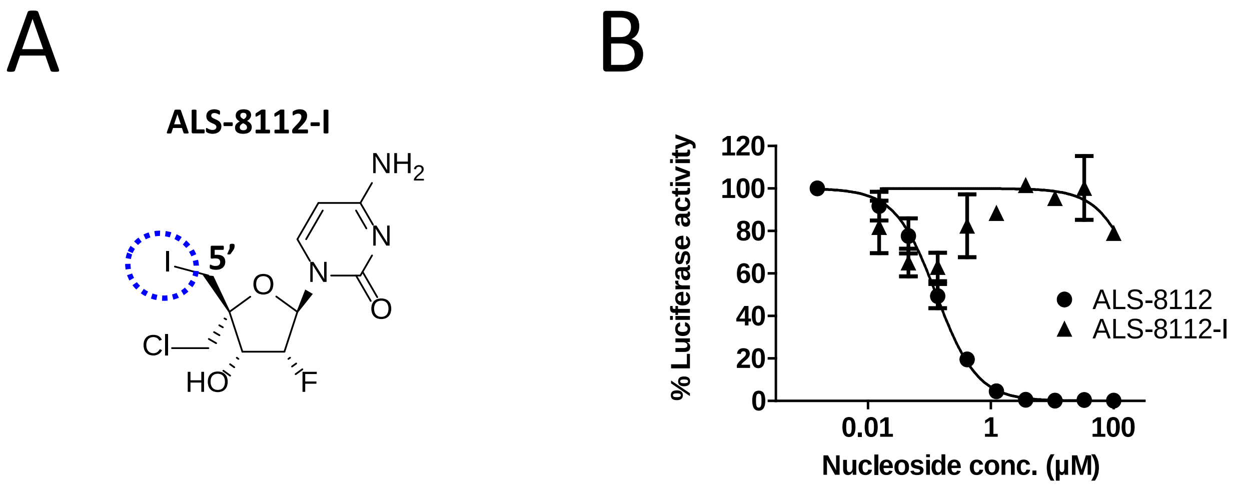
A
ALS-8112-I
NH2
N
I
5'
O
N
O
Cl
HO
F
B
% Luciferase activity
120
100
80
60
40
20
0
0.01
1
100
Nucleoside conc. (μM)
ALS-8112
ALS-8112-I

Figure B

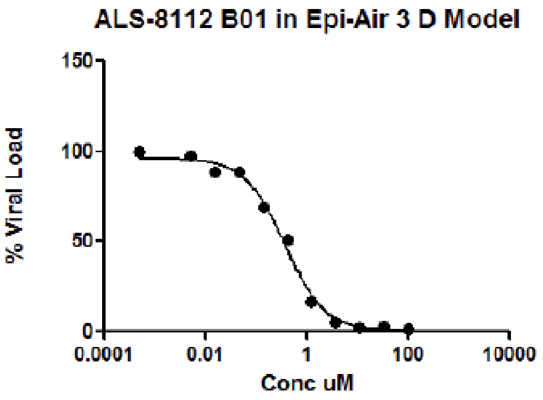

Experiment 1: IC_{50} = 0.37 µM; IC_{90} = 2.7 µM; Donor: #9831, 23-year-old Caucasian male, non-smoker.

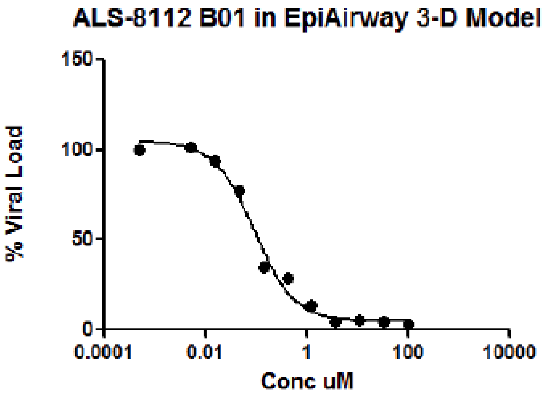

Experiment 2: IC_{50} = 0.09 µM; IC_{90} = 1.3 µM; Donor: TBE-5, 5-year-old Caucasian female.

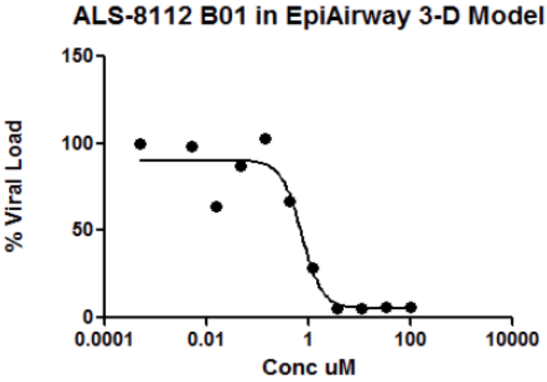

Experiment 3: IC_{50} = 0.73 µM; IC_{90} = 2.7 µM; Donor: TBE-13, 22-month-old Caucasian male, Asthma diagnosed from 5 months of age. Medication: Albuterol.

Figure C

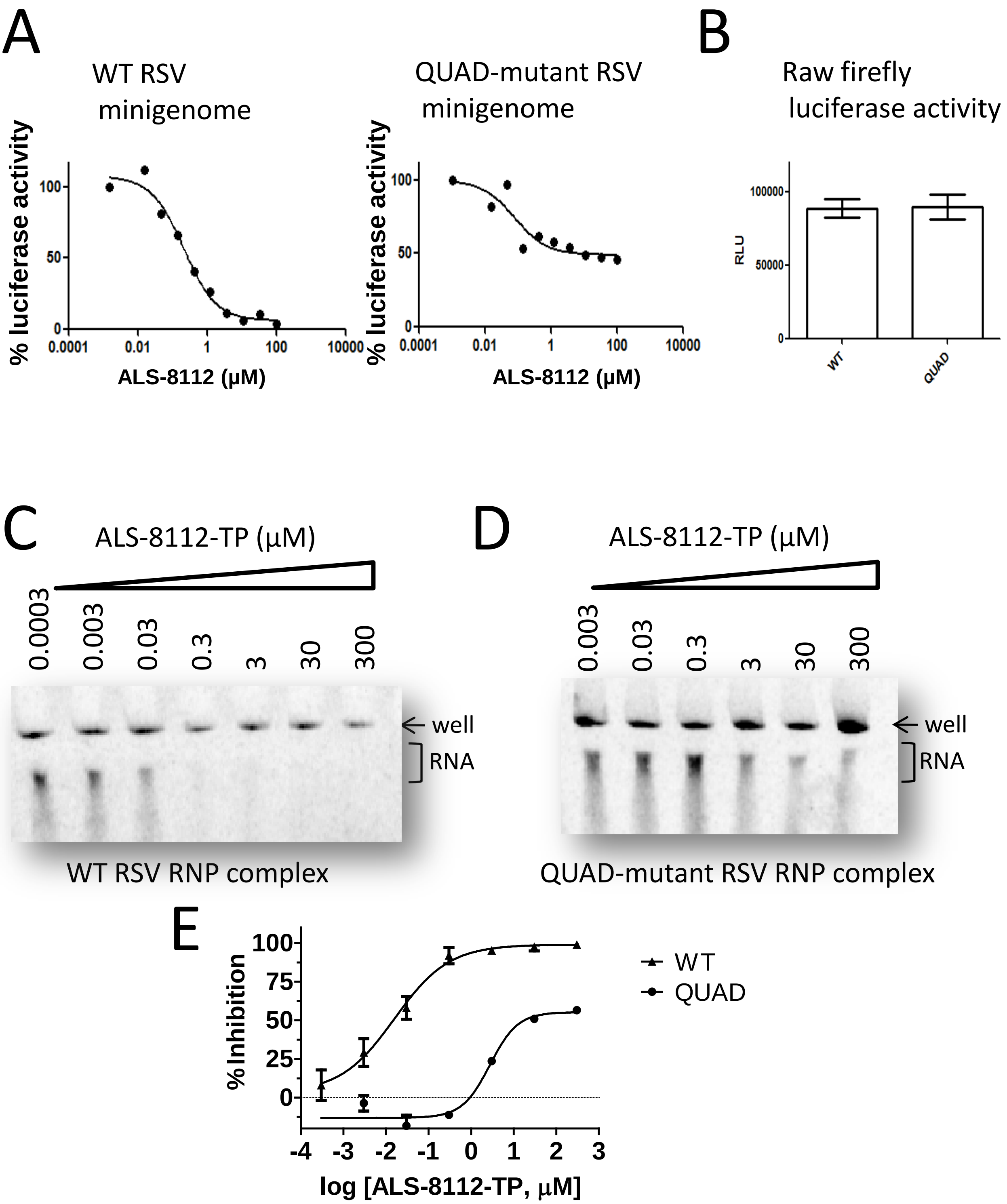

A
WT RSV minigenome
% luciferase activity
100
50
0
0.0001
0.01
1
100
10000
ALS-8112 (µM)
QUAD-mutant RSV minigenome
% luciferase activity
100
50
0
0.0001
0.01
1
100
10000
ALS-8112 (µM)
B
Raw firefly luciferase activity
RLU
100000
50000
0
WT
QUAD
C
ALS-8112-TP (µM)
0.0003
0.003
0.03
0.3
3
30
300
well
RNA
WT RSV RNP complex
D
ALS-8112-TP (µM)
0.003
0.03
0.3
3
30
300
well
RNA
QUAD-mutant RSV RNP complex
E
% Inhibition
100
75
50
25
0
-4
-3
-2
-1
0
1
2
3
log [ALS-8112-TP, µM]
WT
QUAD

Figure D

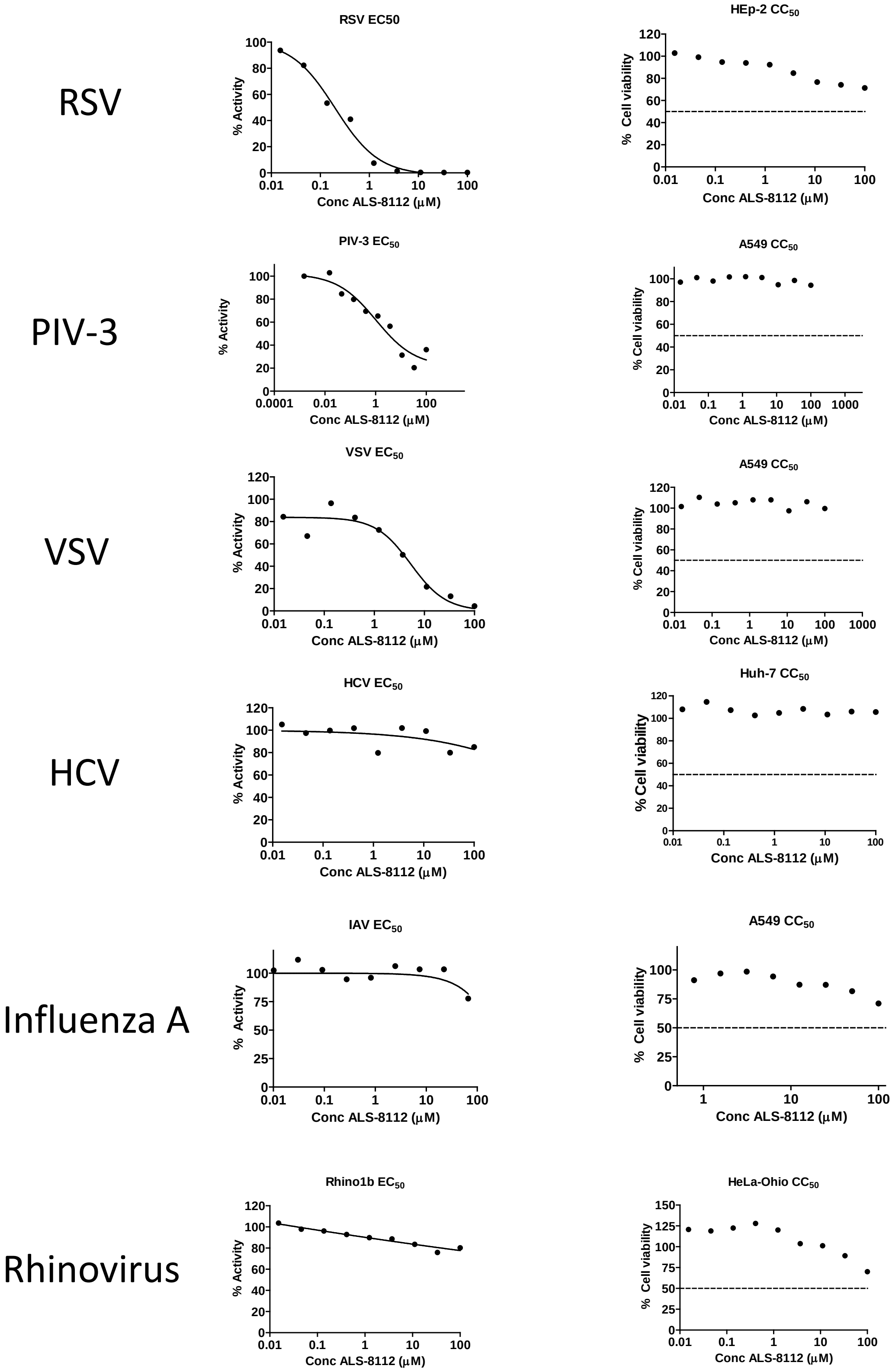
RSV
PIV-3
VSV
HCV
Influenza A
Rhinovirus
RSV EC50
HEp-2 CC50
PIV-3 EC50
A549 CC50
VSV EC50
A549 CC50
HCV EC50
Huh-7 CC50
IAV EC50
A549 CC50
Rhino1b EC50
HeLa-Ohio CC50
% Activity
% Cell viability
Conc ALS-8112 (μM)

Figure E

HCV polymerase

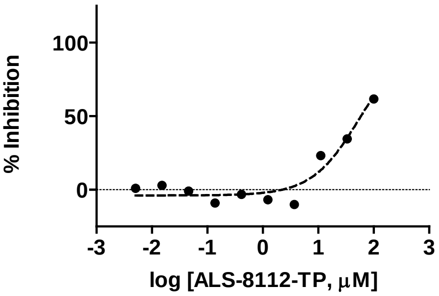

PIV-1 polymerase

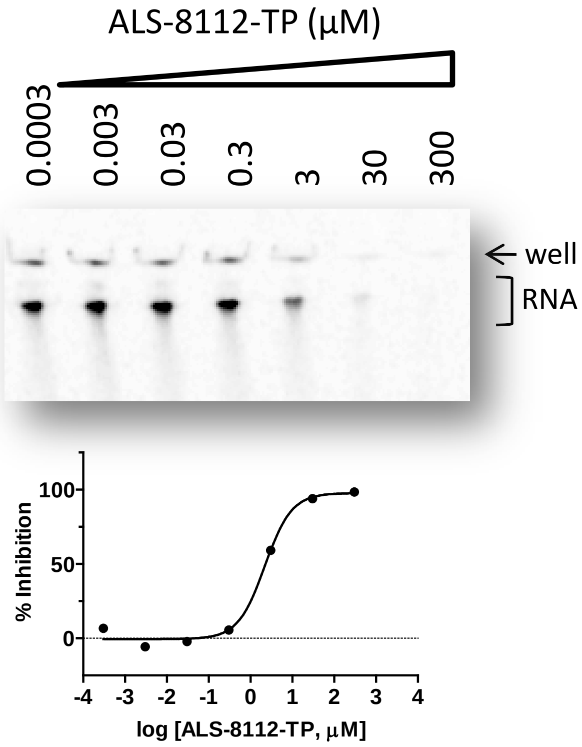

IAV polymerase

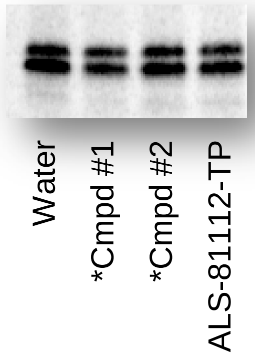

ALS-8112-TP IC_{50} > 100 μM

20.5% inhibition at 100 μM

*unrelated compounds

Figure F

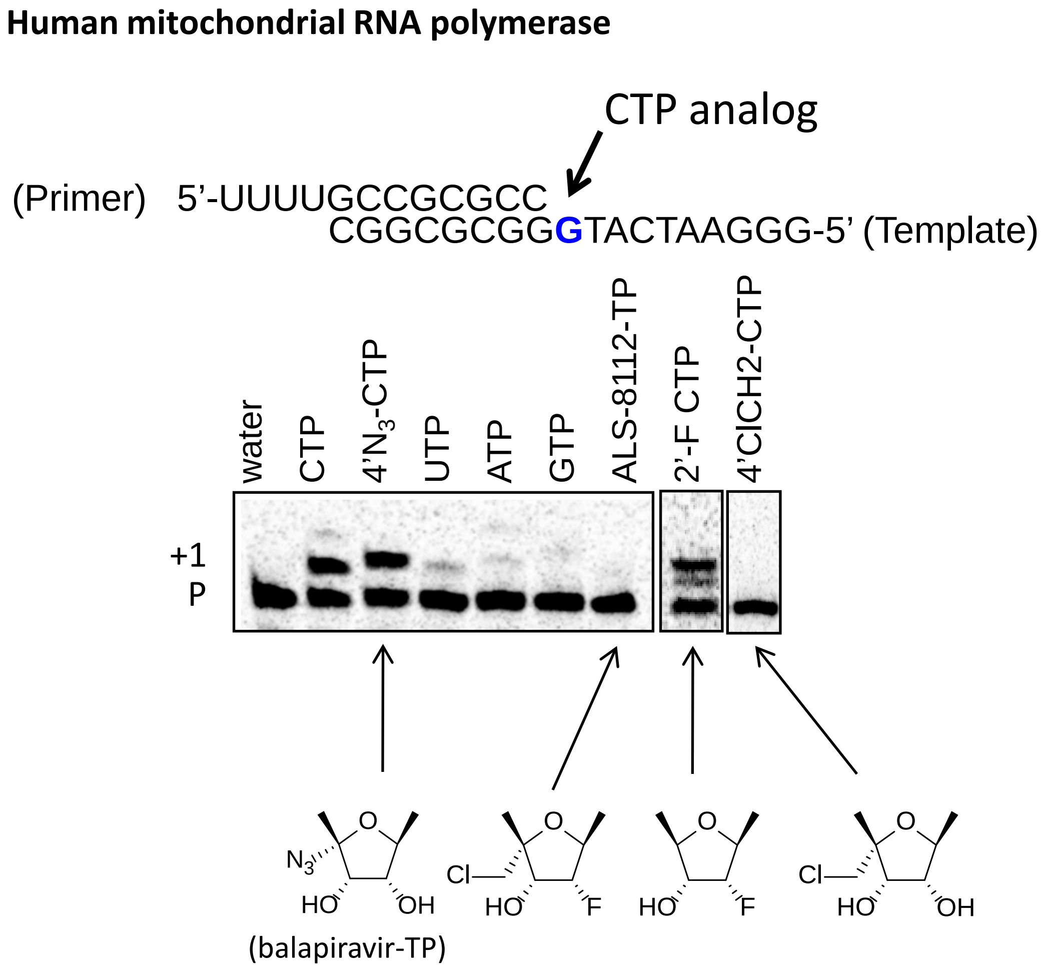

Human mitochondrial RNA polymerase
CTP analog
(Primer) 5'-UUUUGCCGCGCC
CGGCGCGGGTACTAAGGG-5' (Template)
water
CTP
4'N3-CTP
UTP
ATP
GTP
ALS-8112-TP
2'-F CTP
4'ClCH2-CTP
+1
P
N3
HO
OH
O
Cl
F
(balapiravir-TP)

Figure G

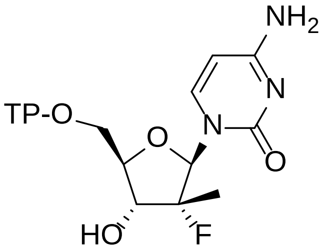

Mericitabine-TP

2'F-2'-Me-CTP

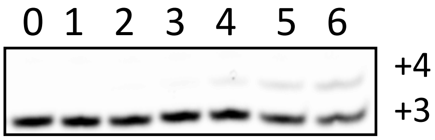

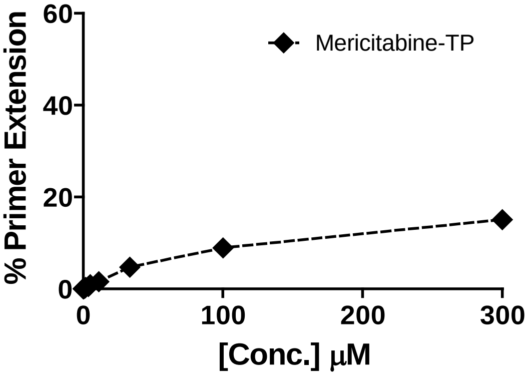

Figure H

A

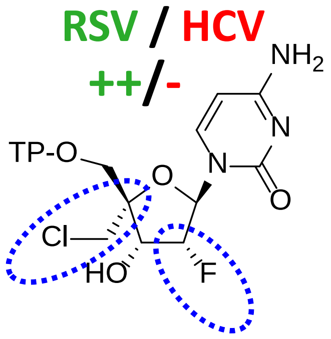

- ++ $IC_{50} < 0.1$ µM
- + $0.1 > IC_{50} > 10$ µM
- - $IC_{50} > 10$ µM

ALS-8112-TP
(2'F-4'ClCH$_2$-CTP)

B

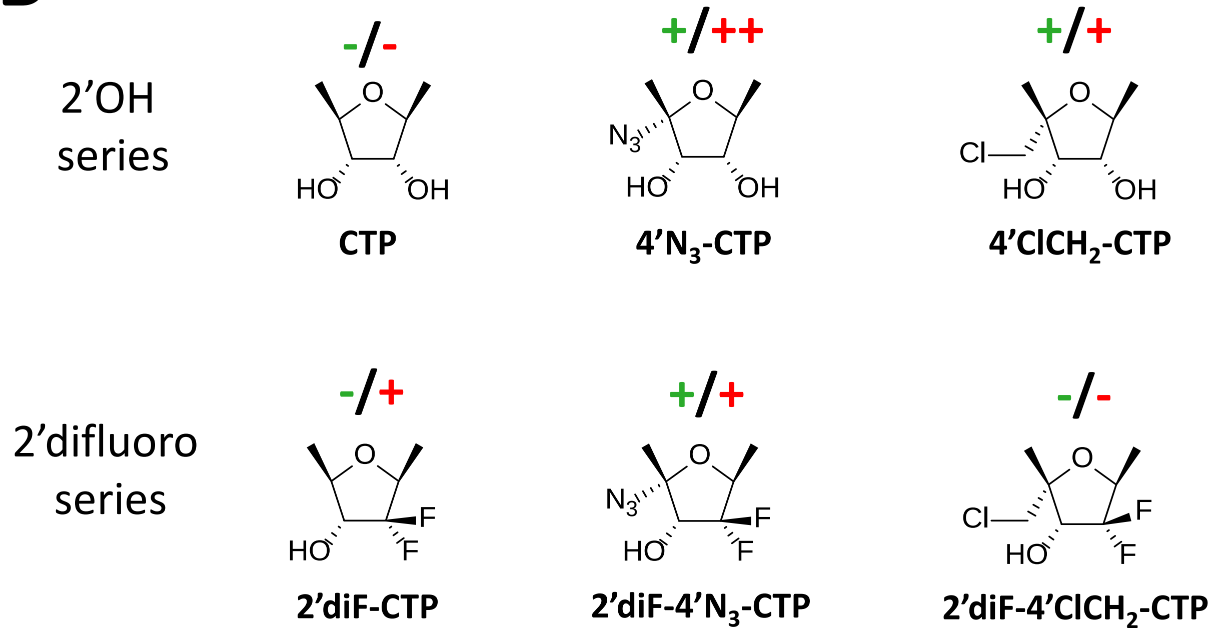

Figure I

A

Calculated Binding Metrics for Nucleotide Analogs

	CDP	2'F-4'ClCH$_2$-CDP (ALS-8112-DP)	2'F-4'N$_3$-CDP	2'F-2'Me-CDP
RMSD, Å	0.00	0.68	0.56	0.51
# H-bonds to Catalytic H_2O	1	0	1	1
# H-bonds to Template	2	1	3	2

B

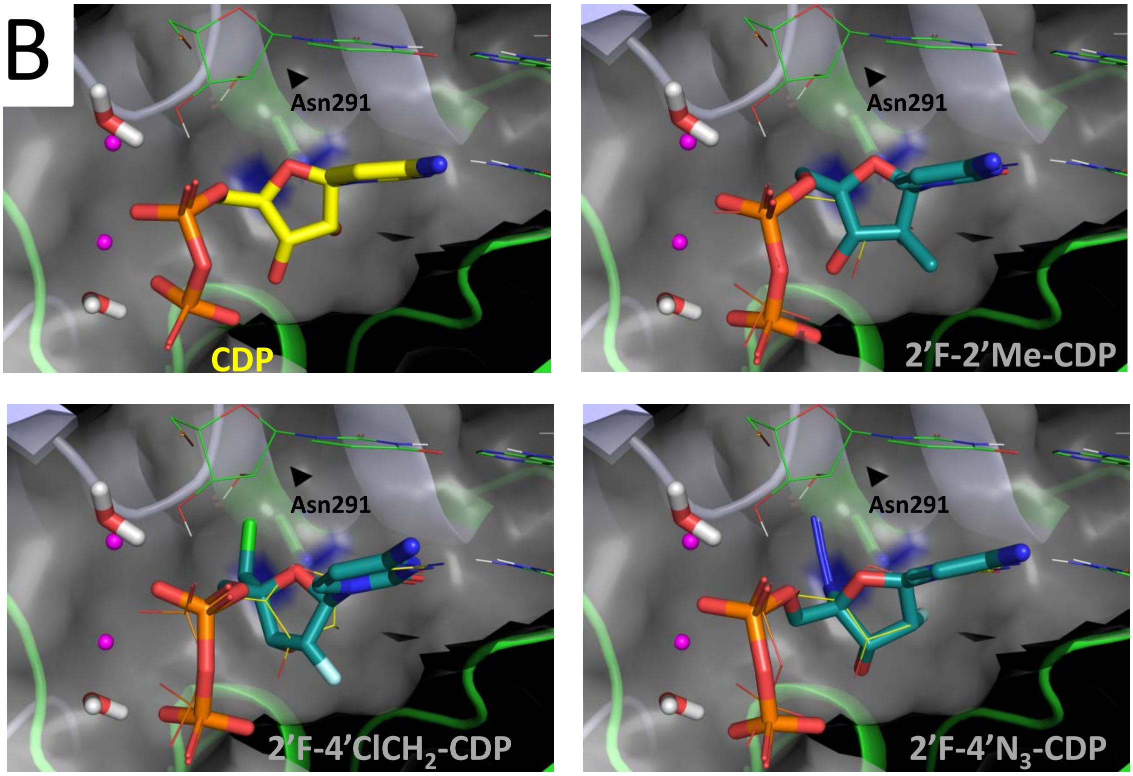

Figure J

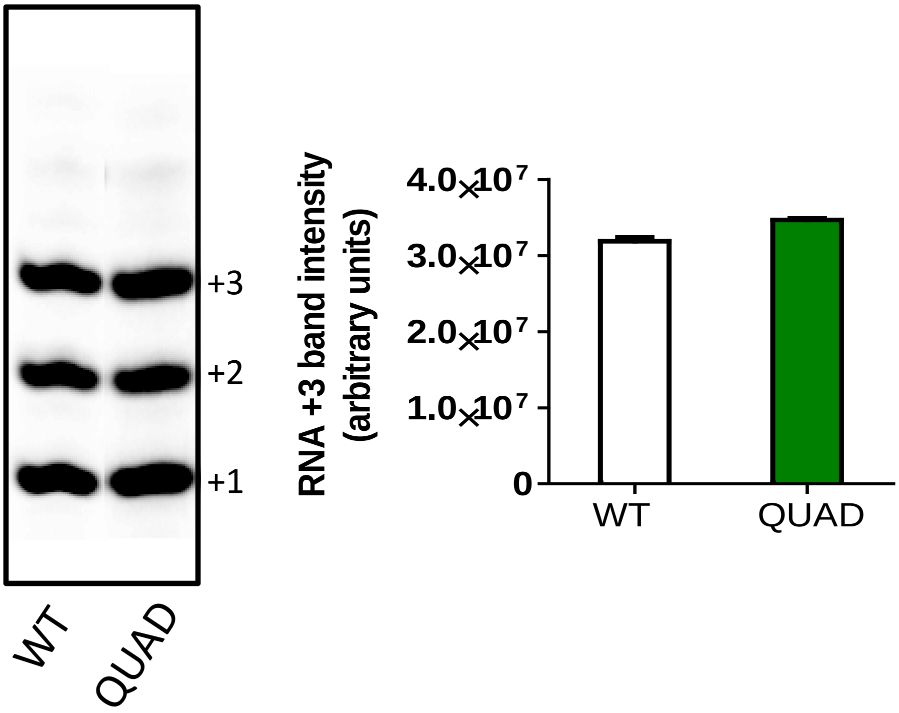
+3
+2
+1
WT
QUAD
RNA +3 band intensity
(arbitrary units)
4.0×10⁷
3.0×10⁷
2.0×10⁷
1.0×10⁷
0
WT
QUAD
